# Effects of Naturalistic Psychedelic Use on Depression, Anxiety, and Well-Being: Associations With Patterns of Use, Reported Harms, and Transformative Mental States

**DOI:** 10.3389/fpsyt.2022.831092

**Published:** 2022-03-15

**Authors:** Charles L. Raison, Rakesh Jain, Andrew D. Penn, Steven P. Cole, Saundra Jain

**Affiliations:** ^1^Department of Human Development and Family Studies, School of Human Ecology, University of Wisconsin-Madison, Madison, WI, United States; ^2^Department of Psychiatry, Texas Tech University Health Sciences Center Medical School, Lubbock, TX, United States; ^3^School of Nursing, University of California, San Francisco, San Francisco, CA, United States; ^4^Research Design Associates, Yorktown Heights, NY, United States; ^5^School of Nursing, University of Texas at Austin, Austin, TX, United States

**Keywords:** psychedelics, depression, anxiety, well-being, psilocybin, ayahuasca, harms, patterns of use

## Abstract

Survey-based studies suggest naturalistic psychedelic use provides mental health benefits similar to those observed in clinical trials. The current study sought to confirm these findings in a large group of psychedelic users and to conduct a novel examination of associations between amount of psychedelic use and behavioral outcomes, as well as frequency of harms ascribed to psychedelic use. A cross-sectional, online survey was completed by 2,510 adults reporting at least one lifetime psychedelic experience. Participants retrospectively completed a battery of instruments assessing depression, anxiety, and emotional well-being prior to and following psychedelic exposure. Participants also reported preferred psychedelic agent, number of uses, and harms attributed to psychedelic use. Psychedelic use was associated with significant improvements in depressive and anxious symptoms and with increased emotional well-being. These improvements increased in magnitude with increasing psychedelic exposure, with a ceiling effect. However, improvements were noted following a single lifetime use. Strong evidence for benefit of one preferred psychedelic agent over another was not observed, but enduring increases in factors related to mystical-experience and prosocial perspective taking associated with enhanced mental health. Thirteen percent of the survey sample (*n* = 330) endorsed at least one harm from psychedelic use, and these participants reported less mental health benefit. Results from the current study add to a growing database indicating that psychedelic use—even outside the context of clinical trials—may provide a wide range of mental health benefits, while also posing some risk for harm in a minority of individuals.

## Introduction

Illegal and long stigmatized as drugs of abuse, psychedelics seem unlikely candidates for the explosion of cultural interest and commercial development they have engendered in recent years. Indeed, as recently as 2015, psychedelics were the sole province of three small organizations working largely out of the public view to bring these agents to market on a non-profit basis. Only a psychic could have foreseen that within 6 years a legion of publicly traded companies—several valued at more than a billion dollars—would have emerged and entered the psychedelic development space to jostle for early supremacy in a market estimated to be eventually worth more than sixteen billion dollars per year (1, 2).

Several factors have converged to fuel this psychedelic gold rush. First and foremost, a handful of small academic studies conducted in the last decade report that psychedelics—when administered in a clinical setting with psychotherapeutic support—produce remarkably rapid, robust, and sustained improvements in a variety of psychiatric and addictive conditions. These conditions include major depressive disorder (3–7), clinically-significant depressive and anxious symptoms in the context of life-threatening cancer (8–11), obsessive compulsive disorder (12), alcohol use disorder and smoking cessation (13–15).

These findings, while preliminary, have generated such excitement in part because their implications stand in sharp contrast to results from a series of landmark studies conducted over the last 20 years that have increasingly eroded confidence in the effectiveness of standard psychopharmacological agents, especially widely used antidepressant medications (16). The current passion for psychedelics may also be driven by a widespread perception that psychedelic treatment by its very nature will demand a longed for, but previously unattainable, integration of pharmacology and psychotherapy (17), as well as by the fact that many people have used psychedelics in naturalistic settings and therefore have firsthand experience of their potential psychological impact.

Despite the current cultural and commercial enthusiasm for psychedelics, currently available data from clinical trials leave many questions of central importance unanswered. Fortunately, a growing database from prior large-scale survey-based studies have provided insight into a wide range of associations either unexplored or only hinted at in clinical trials conducted to date (18). Taken as a whole, these studies provide convergent support for findings from clinical trials, including that psychedelic use (either lifetime or prospective) is associated with increased emotional well-being (19–26), reduced harmful substance use/misuse (i.e., illicit drugs/tobacco/alcohol) (27, 28), a tendency toward liberal political views and an enhanced sense of connection with nature (29, 30). These effects are reliably associated with the occurrence of various types of transformative mental states (e.g., mystical, emotional breakthrough, insight-type) during the acute psychedelic experience that have also predicted outcomes in clinical trials (6, 9, 11, 22, 31–33).

However, given the focus of commercial and non-profit entities on major depressive disorder and generalized anxiety disorder, it is surprising that to our knowledge few large-scale survey studies have used a validated scale to examine whether naturalistic psychedelic use associates with reduced symptoms of major depressive disorder or generalized anxiety disorder in addition to enhanced well-being (19, 23, 26), and these studies comprised significantly smaller sample sizes than the current study. Similarly, no study to our knowledge has examined associations between number of lifetime psychedelic experiences and changes in depressive and anxious symptoms. Relatedly, current commercial and non-profit development programs for psilocybin as a treatment for depression seek approval for a single dose indication (34), raising the question of the degree to which a single lifetime psychedelic exposure might compare in efficacy with more frequent patterns of use. Finally, because significant adverse events have been rare in clinical trials it remains difficult to judge potential associations between negative responses and diminished therapeutic benefit. With the caveat that the negative outcomes seen with naturalistic use might diverge from those observed in clinical settings, would negative outcomes be more frequent in naturalistic settings and might this provide insight into factors that predict lack of therapeutic response to psychedelics?

Although subject to limitations inherent in cross-sectional surveys and with a recognition that the benefits and harms of psychedelic use in a naturalistic environment are likely to be different than those seen in clinical trials, the *Psychedelics and Wellness Study (PAWS)* was designed to address these and other gaps in the psychedelic literature. In particular we sought to test the hypothesis that a robust interrelationship would be observed between past psychedelic use and current levels of emotional wellness (assessed as well-being and depressive and anxious symptoms). We sought to examine associations between frequency of psychedelic use and wellness-related outcomes, as well as the prevalence and types of harms engendered by past use.

## Materials and Methods

### Participant Recruitment and Enrollment

Potential participants were recruited through free online platforms, social media, word-of-mouth, in-person, flyers/postcards, email, and snowball sampling (e.g., referrals from participants to others in their social networks). Online advertisements were targeted for psychedelic specific groups on Facebook, Reddit, and LinkedIn. Recruitment flyers were also posted in community public spaces where permitted. These recruitment materials guided participants to the study's website at www.psychedelicsandwellness.com. To enter the study, potential participants answered inclusion questions related to age (18 and older) and use of psychedelics (at least one time). Exclusion criteria were age younger than 18 and no past history of self-reported psychedelic use. No other criteria were required for enrollment. For participants who met these criteria, the website provided an online consent form that outlined the study's purpose and design, data to be collected, confidentiality standards, and risks and benefits. Upon signing consent, participants were directed to the online survey.

### Study Design

The PAWS Study used an online platform to deliver a cross-sectional survey instrument designed to assess participants' retrospective perspectives on the mental health effects of classic psychedelic use, as well as predictors of positive and negative outcomes from this use. Because of our focus on classic psychedelic agents (i.e., tryptamines and phenethylamines with a primary mechanism of action believed to be agonism of the serotonin 5HT2A receptor), we did not query 3,4-Methylenedioxymethamphetamine (MDMA), which has a different mechanism of action and tends to produce different acute effects than classic psychedelics. Given the widespread emerging use of ketamine as a “psychedelic-like” agent for the treatment of major depressive disorder, we included this agent in our survey, although we recognize that it is not a classic psychedelic.

The survey asked participants to rate their average depressive, anxious and mental well-being status prior to first psychedelic use and then to repeat these ratings based on their average mental health status after psychedelic use. Depressive symptoms were assessed with the 9-item Patient Health Questionnaire (PHQ-9) (35); anxious symptoms were assessed with the 7-item Generalized Anxiety Disorder Scale (GAD-7) (36); and well-being was assessed with the HERO Wellness Scale (37). As an example of the specific language used for these scales, for the Patient Health Questionnaire (PHQ-9) participants were first asked, “*Please answer the following questions based on how you felt PRIOR TO EVER TAKING a psychedelic in your lifetime.”* After completion of this and the other questionnaires, patients were subsequently asked, “*Please answer the following questions based on your overall mood AS A RESULT of your psychedelic experience(s).”*

Following completion of these questionnaires, participants were asked to complete a 26-item battery of questions [26-item Psychedelic Change Questionnaire (PCQ-26)] created specifically for this study to assess change in variables related to the psychedelic experience or to improved or worsened mental health/well-being as a result of that (those) experience(s). Designed to reflect the structure of the Clinical Global Impression Scales, each item offered seven response options ranging from “very much improved” to “very much worse.”

### Survey Instruments and Questions

#### Participant Demographics, Psychedelic Use, and Preferences

The PAWS survey anonymously collected participant, age, sex, education level, preferred psychedelic drug (participants were only allowed to choose one agent), estimated number of lifetime psychedelic uses and history of micro-dosing (yes/no). In addition, given the exponential growth of interest in the use of psychedelics for mental illness, participants were asked if they were a healthcare provider who treats psychiatric disorders with medications.

#### Nine-Item Patient Health Questionnaire

The PHQ-9 is a widely used self-report instrument designed to screen for the presence and severity of depression (35). The PHQ-9 queries each of the nine symptoms that comprise major depressive disorder (MDD) in the DSM 5. Response options for each item range from “*not at all”* to “*nearly every day.”* Scores range from 0 to 27. A cut-off score ≥ 10 shows good sensitivity and specificity for a diagnosis of MDD. Cut-off scores for mild, moderate, moderately severe, and severe depression are 5, 10, 15, and 20, respectively. The PHQ-9 has good internal reliability, with a Cronbach's alpha of 0.89 in the PHQ Primary Care Study (35). Construct validities were 0.73 for mental health, 0.55 for general health perceptions, 0.52 for social functioning, 0.43 for role functioning, 0.37 for physical functioning, and 0.33 for bodily pain.

#### Seven-Item Generalized Anxiety Disorder Scale

The GAD-7 is a self-report instrument widely used for screening and assessment of symptom severity of generalized anxiety disorder (36). GAD-7 items include: (1) nervousness; (2) inability to stop worrying; (3) excessive worry; (4) restlessness; (5) difficulty in relaxing; (6) easy irritation; and (7) fear of something awful happening. As with the PHQ-9, response options for each item range from “*not at all”* to “*nearly every day.”* A total score is derived from adding individual item scores, with ≥10 representing a cut-off for a diagnosis of generalized anxiety disorder that has good sensitivity and specificity (36). Scores of 5-9 constitute mild symptoms, 10-14 indicate moderate symptoms, and scores >15 constitute severe symptoms. The instrument has an internal consistency of 0.92, with validation in primary care and larger treatment settings in the United States and Germany (38).

#### HERO Wellness Scale

The HERO Wellness Scale is a five-item self-report inventory that uses a single question to query each of the following constructs: happiness, enthusiasm, resilience, optimism and overall mental wellness (37). Each item is scored from 0 (*not at all*) to 10 (*extremely*). The HERO Wellness Scale shows good internal consistency (Cronbach's alpha for composite score = 0.93) with adequate corrected item-total correlations (0.67 for resilience to 0.86 for overall mental wellness). The HERO Wellness Scale has been shown to be sensitive to improvements in mental health following a behavioral intervention in patients with psychiatric disorders (39).

#### Twenty Six-Item Psychedelic Change Questionnaire

The PCQ-26 was created specifically for this study. The PCQ-26 queries a mixture of emotional states that often occur during the psychedelic experience itself, such as a sense of awe, connection with nature and feelings of joy, but queries longer-lasting change in these emotions because of psychedelic use, rather than their occurrence during psychedelic dosing sessions *per se*. In addition, the PCQ-26 queries symptoms common to a variety of mental disorders, such as ruminative thinking and suicidal ideation, as well as substance misuse and criminality. Each item is scored from 1 (Very much improved) to 7 (Very much worse). As described below, exploratory factor analysis revealed three principal components that account for 59% of scale variance. A copy of the PCQ-26 is provided in Supplemental Material.

#### Eight-Item Negative Consequences Inventory

The NCI-8 was created specifically for this study. While recognizing that the universe of potential harms is nearly infinite, the NCI-8 focuses on concerns that have been of primary historic significance for the use of psychedelics; specifically, that these agents would encourage ongoing illicit drug use and lead to problematic/antisocial behavior. The NCI-8 queried eight potential negative outcomes participants ascribed to their psychedelic use, including increased suicidal desire, criminal impulses/behaviors, aggressive impulses/behaviors, alcohol misuse, cigarette smoking, cannabis/marijuana misuse, benzodiazepine misuse, and opiate/opioid misuse. Each item is scored from 1 (Very much improved) to 7 (Very much worse). A copy of the NCI-8 is provided in Supplemental Material.

The entire PAWS survey is available as supplemental information to this article.

### Ethical Considerations

The PAWS study was conducted on a completely anonymous basis, with no personally identifying data collected. All relevant items queried past psychedelic use with no assessment of any potential future psychedelic use. The survey provided no endorsement of psychedelic use, and participants were not compensated. Given its minimal risk status (e.g., conducting a survey with de-identified participants asking about past behavior), the Western Institutional Review Board (WIRB) determined the study to be exempt under 45 CFR § 46.104(d) (2). The PAWS study was registered on Clinicaltrials.gov (ID: NCT04040582).

### Statistical Analyses

Frequency distributions were calculated for all measures and means, and standard deviations were computed for all continuous measures. Distributions of the outcome measures were examined for outliers and for significant deviations from normality. For post and post-pre difference scores for PHQ-9, GAD-7, and HERO measures, Kolmogorov-Smirnoff and Shapiro Wilk tests indicated significant deviations from normality. However, bootstrap simulations based on 500 samples demonstrated that the underlying distributions were normally distributed (Kolmogorov-Smirnoff and Shapiro-Wilk tests *p* > 0.05), indicating that use of parametric tests was appropriate.

To optimize its use in subsequent analyses, exploratory factor analysis (EFA) was applied to the PCQ-26 to examine potential underlying structures and to reduce dimensionality and the corresponding risk for type I error. To determine the number of factors to be extracted, we performed a Monte Carlo simulation of normal random samples that parallel the observed data in terms of sample size and number of variables used. This parallel analysis served as a comparison against the observed eigenvalues. Following standard procedure, loading scores were categorized as follows: >0.71 (50% overlapping variance) as excellent; 0.63-0.71 as very good; 0.55-0.62 as good; 0.45-0.54 as fair; and 0.32-0.44 as poor (40). Items that cross-load onto more than one factor were considered significant if the difference in loading scores is ≥0.2. As described in the Results section, three factors were identified and included as predictors in a regression model for each of the primary outcomes as dependent variables.

Paired sample *t*-tests were used to compare PHQ-9, GAD-7 and HERO scores prior to (pre), and following (post), psychedelic use. Effect sizes for these comparisons were expressed as Cohen's *d*. Effect sizes from ≥0.2 to <0.5 are considered small; effect sizes from ≥0.5 to <0.8 are considered medium; and effect sizes ≥ 0.8 are considered large (41). To evaluate variables (e.g., demographic variables, PCQ-26 factors) that might impact primary outcomes, linear regressions were run on PHQ-9, GAD-7 and HERO residualized change scores. Using regression line equations, predicted scores were calculated for each participant, after which a residual was calculated for each participant (e.g., post score minus the predicted score). The residual scores were standardized so that the mean of the residuals = 0 with a standard deviation = 1.0. This residual change measure was used as the dependent variable for multiple regressions in which scores on variables of interest were used as predictor variables. This strategy allowed us to estimate the association between a given predictor variable and the outcome holding all other variables constant, thereby providing a method of adjusting for potential confounding variables that have been included in the model. Standardized beta coefficients were used to compare the strength of the effect of each individual predictor variable on the dependent variable.

Statistical significance was set at an alpha <0.05 (two-tailed). Analyses were conducted using SPSS version 27 (IBM Corp, Armonk, NY).

## Results

### Demographics

Table 1 presents demographic information on the 2,510 adults who completed the study survey. Participants ranged in age from 18 to 86, with an equal representation of males and females. Fifty-eight percent had a bachelor's degree or higher. The study sample averaged 38.55 (range 1-500) lifetime uses of a psychedelic, with most participants identifying psilocybin or LSD as their preferred psychedelic (psilocybin 51.6%; LSD 30.1%). Ninety participants (3.6%) reported a single use.

**Table 1:** Sample characteristics (*N* = 2,510).

**Variables**	***N***	**%**	**M**	**SD**	**Range**
Age (yrs)	2,510		35.17	14.33	18-86
**Gender**					
Female	1,222	48.7			
Male	1,253	49.9			
Other	35	1.4			
**Education**					
Less than high school	43	1.7			
High school or equivalent	270	10.8			
Some college	573	22.8			
Associate degree	166	6.6			
Bachelor's degree	705	28.1			
Graduate degree	479	19.1			
Professional degree	274	10.9			
**Preferred psychedelic**					
Psilocybin (magic mushrooms)	1,295	51.6			
LSD	755	30.1			
Ayahuasca	145	5.8			
DMT	80	3.2			
Ketamine	69	2.7			
Mescaline/peyote/San Pedro/other mescaline containing cacti	51	2.0			
Other designer/synthetic	46	1.8			
5-MeO-DMT	42	1.7			
Salvia	10	0.4			
lboga/Ibogaine	8	0.3			
2C-B	7	0.3			
2C-E	2	0.1			
**Total number of times taken any psychedelic in lifetime**	2,510		38.55	76.15	1-500
**Ever micro-dosed a psychedelic substance**					
Yes	1,525	60.8			
No	985	39.2			
**Healthcare provider**					
Yes	207	8.2			
No	2,303	91.8			

### Association of Lifetime Psychedelic Use With Depression, Anxiety and Emotional Well-Being

As shown in Table 2, survey respondents reported that their use of psychedelics was associated with significant reductions in depressive and anxious symptoms and increases in emotional well-being. Based on retrospective self-report, PHQ-9 and GAD-7 scores dropped, and HERO-assessed wellness scores increased from average values prior to any lifetime psychedelic use to average values post-psychedelic exposure [PHQ-9: *t*_(2,509)_ = 51.54, *p* < 0.001; GAD-7: *t*_(2,509)_ = 52.79, *p* < 0.001; HERO: *t*_(2,509)_ = 53.73, *p* < 0.001]. These results represent large pre- to post-exposure effect size changes (PHQ-9: *d* = 1.07; GAD-7: *d* = 1.10; HERO: *d* = 1.07).

**Table 2:** Depression, anxiety, and well-being scores for pre- and post-psychedelic usage.

	**Pre**		**Post**		
**Measure**	**Mean**	**SD**	**Mean**	**SD**	***t***	***p***	**Cohen's *d***
PHQ-9	10.70	6.62	4.65	4.35	51.54	<0.001	1.07
GAD-7	9.33	5.91	3.59	3.88	52.79	<0.001	1.10
HERO	27.99	10.88	39.31	7.55	53.73	<0.001	1.07

*PHQ-9, Patient Health Questionnaire*.

*GAD-7, Generalized Anxiety Disorder Scale*.

*HERO, HERO Wellness Scale*.

### PCQ-26: Factor Analysis and Association With Lifetime Psychedelic Use

Of the 26 PCQ items, only item 5 “Relationship with your life partner” had missing data. Seven hundred and eighty five of the 2,510 respondents (31.3%) considered this question to be inapplicable to their life situation. To include applicable data for item 5, a mean substitution of missing data procedure was used. Exploratory factor analysis identified three factors with eigenvalues ≥ 1, which together accounted for 59.6% of the scale variance. The ratio between the first and second eigenvalues was high (9.1), with Factor 1 accounting for 49.8% of the variance; Factor 2 accounting for 5.5% of the variance; and Factor 3 accounting for 4.4% of the variance. The factors were moderately correlated with each other. The highest correlation was between Factors 1 and 2 (*r* =.65) and the lowest was between Factors 2 and 3 (*r* = 0.44). A Monte-Carlo simulation of normal random samples confirmed the appropriateness of a three-factor solution.

The PCQ-26 items that loaded onto each factor are presented as a pattern matrix in Table 3. Item loadings > 0.32 with differences in cross-loaded values ≥ 0.2 were interpreted. There were 13 such items for Factor 1 with Cronbach's α = 0.94; 6 items for Factor 2, Cronbach's α = 0.83; and 3 items for Factor 3, Cronbach's α = 0.82. Interestingly, although assessed as enduring perceptions/cognitions/emotions in the PAWS survey, most Factor 1 items are frequently endorsed as being experienced during the acute psychedelic experience itself, often under monikers such as “mystical-type,” “peak,” “unitary,” or “transformative.” Factor 2 items overlap with emotional and physical states/functions, such as irritability, rumination, sleep, appetite, and sexuality, that are reliably altered in depressive/anxious conditions. Factor 3 items unanimously reflect prosocial emotions and motivations. Because the scoring for each item was 1 = very much improved and 7 = very much worse, a lower factor score indicates a higher value for the construct. For example, a respondent with a negative score for Factor 3 would have more improved philanthropic desire, desire for world peace, altruistic desire, feelings of empathy and feelings of compassion than would a participant with a more positive score.

**Table 3:** Factor loadings for PCQ-26.

**Item**	**Item no**.	**Factor 1**	**Factor 2**	**Factor 3**
Connection to the universe	10	**0.879**	−0.270	0.141
Connection to nature	11	**0.874**	−0.271	0.217
Sense of awe	1	**0.779**	−0.192	0.111
Feelings of inner peace	8	**0.761**	0.305	−0.233
Sense of mindfulness	4	**0.699**	0.001	0.091
Feelings of joy	13	**0.670**	0.190	0.024
Feelings of openness	14	**0.648**	0.023	0.140
Sense of calm	9	**0.644**	0.390	−0.209
Feelings of contentment	15	**0.630**	0.280	−0.078
Feelings of gratitude	16	**0.621**	0.026	0.237
Enjoyment of life	20	**0.619**	0.250	0.004
Feelings of love	12	**0.586**	0.075	0.262
Feelings of social connectedness	3	**0.525**	0.123	0.090
Sense of purpose	17	0.433	0.266	0.132
Quality of sleep	7	−0.224	**0.817**	0.123
Eating habits	25	−0.260	**0.697**	0.319
Feelings of irritability	26	0.143	**0.695**	0.004
Ruminative thinking	23	0.301	**0.632**	−0.177
Feelings of sexual intimacy	6	−0.084	**0.539**	0.291
Relationship with life partner	5	0.191	**0.447**	0.153
Fear of death	24	0.232	0.240	0.224
Philanthropic desire	22	−0.108	0.283	**0.720**
Desire for world peace	19	0.132	0.040	**0.685**
Altruistic desire	21	0.243	0.048	**0.649**
Feelings of empathy	2	0.400	−0.062	0.502
Feelings of compassion	18	0.391	0.092	0.485

*Factor loading values are regression coefficients from the pattern matrix*.

*Loadings in bold font are ≥0.32 with differences in cross-loaded values ≥ 0.2*.

To determine the impact of psychedelic usage on each of the PCQ-26 factors we scored the cumulative percentage of participants entering minimally improved, much improved, and very much improved responses for each item and then averaged these responses. As shown in Table 4, based on this method, 91.7% reported improvements on Factor 1; 66.2% reported improvements on Factor 2; and 77.8% reported improvements on Factor 3.

**Table 4:** Percentage of respondents indicating improvement on factor items.

**Item**	**Item no**.	**Factor 1**	**Factor 2**	**Factor 3**
Connection to the universe	10	93.7		
Connection to nature	11	93.9		
Sense of awe	1	95.4		
Feelings of inner peace	8	92.9		
Sense of mindfulness	4	94.1		
Feelings of joy	13	89.9		
Feelings of openness	14	92.8		
Sense of calm	9	89.1		
Feelings of contentment	15	87.7		
Feelings of gratitude	16	90.4		
Enjoyment of life	20	91.8		
Feelings of love	12	90.5		
Feelings of social connectedness	3	89.5		
	Mean 91.7			
Quality of sleep	7		52.5	
Eating habits	25		49.4	
Feelings of irritability	26		72.9	
Ruminative thinking	23		78.8	
Feelings of sexual intimacy	6		62.5	
Relationship with life partner	5		81.0	
		Mean 66.2		
Fear of death	24			70.0
Philanthropic desire	22			77.3
Desire for world peace	19			86.1
			Mean 77.8	

### Negative Outcomes Associated With Lifetime Psychedelic Use

The NCI-8 queried participants regarding harms they may have experienced as a result of psychedelic use, with these harms being divided between behavioral disturbance (e.g., suicidal desire, criminal behavior) and substance misuse. Table 5 shows the counts for participants who responded to an increasing number of NCI items with “minimally worse” through “very much worse.” Altogether, 330 participants (13%) endorsed at least one negative outcome they attributed to psychedelic use, and some participants endorsed multiple negative outcomes, leading to a total of 476 negative item responses. Table 6 shows the relative frequency of each of the eight negative items in the population of participants who endorsed at least one negative outcome.

**Table 5:** Number and percentages of negative responses.

**Number of responses**	**Frequency**	**Percent**	**Cumulative percent**
0	2180	86.9	86.9
1	240	9.6	96.4
2	56	2.2	98.6
3	22	0.9	99.5
4	6	0.2	99.8
5	4	0.2	99.9
6	1	0.0	100.0
7	0	0.0	100.0
8	1	0.0	100.0

**Table 6:** Number and percentages of negative responses to psychedelic use.

**Negative outcome items**	**Response**		**Percent of cases**
	**Number**	**Percent**	
Desire to die by suicide	37	7.8%	11.2%
Criminal impulses/behavior	65	13.7%	19.7%
Aggressive impulses/behaviors	36	7.6%	10.9%
Alcohol misuse	54	11.3%	16.4%
Cigarette smoking	103	21.6%	31.2%
Cannabis misuse	152	31.9%	46.1%
Benzodiazepine misuse	19	4.0%	5.8%
Opiate/opioid misuse	10	2.1%	3.0%

*Responses, out of 476 endorsed negative outcomes reported; Percent of cases, percent out of the 330 participants who endorsed at least one negative outcome*.

**Figure 1:**
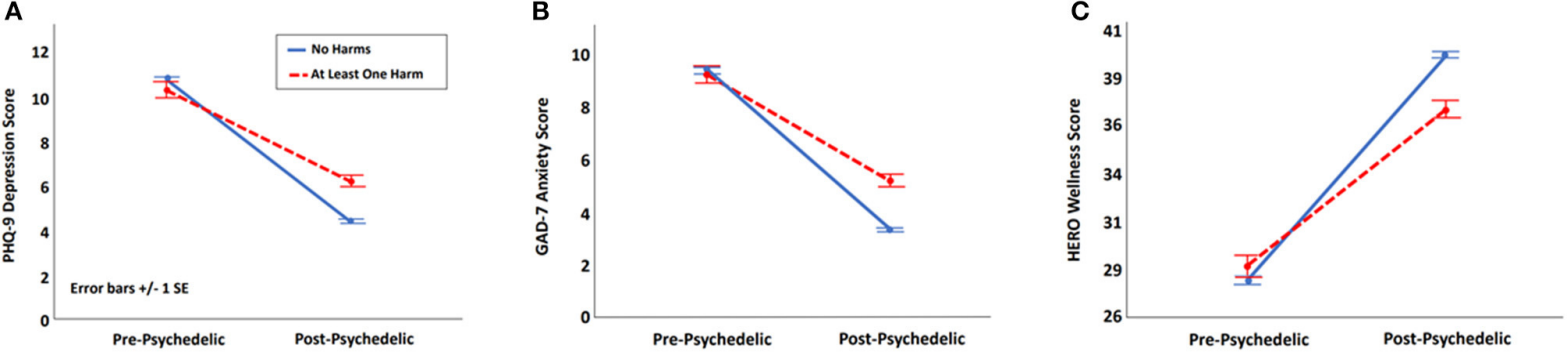
Effect of psychedelic exposure in participants with and without reported harms for use. **(A)** Effect of harms on depressive symptoms measured with the 9-items Patient Health Questionnaire (PHQ-9). **(B)** Effect of harms on anxious symptoms measured with the 7-item Generalized Anxiety Scale (GAD-7). **(C)** Effect of harms on wellness measured with the Hero Wellness Scale.

To explore the effect of negative outcomes on associations between psychedelic use and reductions in depression and anxiety and increases in emotional well-being, we compared PHQ-9, GAD-7, and HERO scores from pre- to post-psychedelic use in participants with one or more negative outcome (*n* = 330) vs. with those with none (*n* = 2,180). As shown in Figure 1 participants with one or more negative outcome derived significantly less benefit from psychedelic use, despite showing no differences in their assessment of pre-psychedelic symptom status [PHQ-9: *t*_(2, 508)_ = 6.55, *p* < 0.001, *d* = 0.39; GAD-7: *t*_(2, 508)_ = 6.27, *p* < 0.001, *d* = 0.37; HERO: *t*_(2, 508)_ = 5.76, *p* < 0.001, *d* = 0.34].

### Associations Between Patterns of Psychedelic Use, Agent of Choice and Change in Depression, Anxiety and Emotional Well-Being

Extent of lifetime psychedelic use was not associated with pre-exposure scores on the PHQ-9, GAD-7, or HERO. Increasing lifetime psychedelic use was associated with larger reductions in scores on PHQ-9 and GAD-7 scales, and larger increases on the HERO scale (PHQ-9: *r* = −0.06, *p* = 0.006; GAD-7: *r* = −0.04, *p* = 0.040; HERO: *r* = 0.10, *p* < 0.001). Curve fitting analyses indicated that associations between lifetime usage and HERO scores were best explained by a sigmoidal curve (*r*^2^ = 0.52, *p* = 0.018) such that benefit increased and then leveled off after approximately five psychedelic exposures. No significant curves were identified for the PHQ-9 or GAD-7. Table 7 presents associations between lifetime use (parceled out in groups of five, e.g., 1-5, 6-10….50+) and mean change score, as well as the effect size of this score for the PHQ-9, GAD-7 and HERO scales.

**Table 7:** Change in PHQ-9, GAD-7, and HERO scores by lifetime psychedelic usage.

		**PHQ-9**		**GAD-7**		**HERO**	
**Number of uses**	***N***	**Mean**	***d***	**Mean**	**d**	**Mean**	**d**
1-5	610	−5.52	0.90	−5.23	0.95	9.20	0.90
6-10	544	−5.88	1.05	−5.55	1.07	9.92	0.99
11-15	273	−6.13	1.05	−5.80	1.11	12.08	1.19
16-20	214	−5.86	0.98	−5.57	1.02	12.63	1.20
21-25	113	−6.62	1.05	−6.17	1.19	13.15	1.14
26-30	148	−6.98	1.16	−6.76	1.32	12.58	1.28
31-35	38	−7.74	1.36	−6.76	1.30	13.74	1.45
36-40	73	−6.82	1.17	−6.07	1.04	12.20	1.16
41-45	9	−9.78	1.74	−7.67	1.87	10.78	1.16
46-50	96	−6.05	0.93	−5.90	0.90	13.33	1.04
>50	392	−6.26	1.06	−6.13	1.09	13.43	1.25

*PHQ-9, Patient Health Questionnaire*.

*GAD-7, Generalized Anxiety Disorder Scale*.

*HERO, HERO Wellness Scale*.

*d, Cohen's d*.

Given that current commercialization efforts for psilocybin as a treatment for MDD propose single-dose regimens, we examined the effect of psychedelic use in the 90 survey respondents who reported only one lifetime use. Although change scores were smaller than for participants with greater lifetime exposure, significant reductions in depression and anxiety and increases in emotional well-being from pre- to post-psychedelic exposure were also evident in this group: PHQ-9: *t*_(89)_ = 6.57, *p* < 0.001, *d* = 0.68; GAD-7: *t*_(89)_ = 6.82, *p* < 0.001, *d* = 0.72; HERO: *t*_(89)_ = 6.99, *p* < .001, *d* = 0.74.

The majority of respondents (81.7%) identified either psilocybin or LSD as the agent they felt had been most beneficial for them. When comparing each of these agents vs. all others, no differences were seen in pre-exposure HERO, PHQ-9 or GAD-7 scores. Similarly, repeated measures ANOVA showed no main or interaction effects for either psilocybin vs. all others (main effects: HERO, *p* = 0.439; PHQ-9, *p* = 0.884; GAD-7, *p* = 0.283; group x time interactions: HERO, *p* = 0.439; PHQ-9, *p* = 0.884; GAD-7, *p* = 0.283) or LSD vs. all others (main effects: HERO, *p* =.175; PHQ-9, *p* =.950; GAD-7, *p* = 0.269; group x time interactions: HERO, *p* = 0.610; PHQ-9, *p* = 0.504; GAD-7, *p* = 0.111). On the other hand, the 145 participants who endorsed ayahuasca as their preferred agent reported enhanced improvements in HERO-assessed well-being vs. all other agents (group x time interaction, *p* = 0.010, effect size, *d* = 0.22); however, no differences were observed for the PHQ-9 or GAD-7. Group x time interactions were also observed for ketamine preference (*N* = 69) vs. all other agents for depressive and anxious symptoms (PHQ-9, *p* = 0.035; *d* = 0.31; GAD-7, *p* = 0.047; *d* = 0.24), with a trend level interaction for wellness (HERO, *p* = 0.063; *d* = 0.22), but these effects were accounted for by the fact that these participants reported lower HERO and higher PHQ-9 and GAD-7 scores pre-psychedelic exposure when compared to participants who preferred other agents. Indeed, PHQ-9 and GAD-7 scores remained higher in these participants following ketamine exposure than in participants who endorsed preferring other psychedelic agents (PHQ-9, *p* = 0.006; GAD-7, *p* = 0.035) and no difference was observed for HERO scores (*p* = 0.255).

### Factors Independently Associated With Change in Depression, Anxiety and Emotional Well-Being

Multiple regression was used to identify demographic, PCQ-26 and patterns of use variables independently associated with change in PHQ-9, GAD-7, and HERO scores from pre- to post-lifetime psychedelic use. PCQ-26 factors 1 and 2 were independently associated with reduced PHQ-9 and GAD-7 scores (*p* < 0.001) and increased HERO scores (*p* < 0.001) indicating reduced depression and anxiety and improved well-being. Participants who reported negative outcomes from psychedelic use also reported less improvement in PHQ-9 and GAD-7 scores (*p* < 0.001).

## Discussion

The current study is, to our knowledge, one of the largest to date to examine self-reported associations between psychedelic use and depression, anxiety, and emotional well-being. Within the constraints imposed by the type of retrospective self-report methodology employed here, current results support our *a priori* hypothesis that a robust interrelationship would be observed between past psychedelic use and current levels of emotional wellness, thereby providing intriguing insights into associations between psychedelic use and mental health. Consistent with findings from clinical trials (3–6, 9–13), and other survey-based studies (19, 23, 26) psychedelic use in our population was associated with large effect size reductions in depression and anxiety and marked improvements in emotional well-being. These benefits increased with self-reported psychedelic usage, but even participants with a single life-time psychedelic exposure reported improvements in mental health. Participants who reported benefitting most from ayahuasca reported enhanced emotional well-being compared to participants who preferred other agents, although the effect size for this advantage was small (*d* = 0.22).

In addition to mental health benefits, most participants reported sustained increases in PCQ-26-assessed transformative/pivotal states of mind/attitudes (42) often observed during dosing sessions in clinical trials (e.g., mystical, emotional breakthrough, insight-type), (6, 9, 11, 22, 31–33) as well as increases in self-perceived altruism and prosocial behavior. To various degrees, these effects were important independent predictors identified for improvements in depression, anxiety, and emotional well-being associated with psychedelic use. However, not all participants reported unqualified benefit from psychedelic usage. Thirteen percent identified at least one harm, and these participants reported receiving significantly less mental health benefit from their psychedelic usage than participants not endorsing any harms.

The current study has a number of limitations that warrant mention, including its retrospective design, reliance on self-report, inability to confirm that respondents actually took the psychedelics as reported or took them the number of reported times. Although common to online retrospective survey-type studies, these limitations suggest that results from the current investigation should be considered as hypothesis generating, rather than confirmatory. Interestingly, a strength of the current study comes from one of its limitations: in this case the fact that the participant sample is self-selected. While this limits our ability to draw conclusions regarding the value of psychedelic exposure for mental health in the general population, it provides a window into relationships between self-reported depression, anxiety and well-being and patterns of psychedelic use in a large population with more extensive (and a wider range of) drug exposure than would be common in a general population survey. In general, therapeutic benefit increased with increasing usage, although this association was not linear. Indeed, for HERO-assessed well-being, benefit increased over the first 5-10 doses and then leveled off in a statistically significant “s-shaped” pattern. Although not significant, numerical improvements in the PHQ-9 and GAD-7 appeared to plateau between 15 and 20 lifetime uses.

These findings may challenge current psychedelic development programs that propose limited dosing strategies, especially given our finding that participants with a single lifetime psychedelic exposure reported significantly less improvement in depression, anxiety and well-being than did those with higher levels of use. However, our study design does not allow us to determine whether associations between increased psychedelic use and enhanced self-reported mental health reflect benefits derived from dose loading strategies (i.e., repeated dosing over a short period of time to maximize acute effects), maintenance of effect strategies (i.e., treatment to prevent or treat relapse) or both. However, if one assumes that these self-reported patterns of use map at least somewhat onto the clinical needs of the study participants, current results suggest that psychedelics may require a significant degree of redosing for maximal therapeutic effect. On the other hand, our findings suggest a point of diminishing returns in regard to the mental health benefits individuals tend to receive from psychedelic use in naturalistic settings. If confirmed in subsequent populations, this will highlight the importance of using psychedelics as spurs to initiate changes in lifestyle/perspective that are mental health protective, rather than attempting to use these agents as lifelong mood modifiers.

Because the PAWS study specifically sought to enroll participants with psychedelic experience and included questionnaires focused on emotional well-being, it is possible that a combination of biased recruitment and demand characteristics inflated improvements in mental health ascribed to psychedelic use. For example, individuals who experienced less perceived benefit from psychedelics would also likely be less interested in completing the PAWS survey, and individuals strongly supportive of psychedelic use (such as would be most likely to enroll) would be most likely to recognize that positive answers might allow the survey to support the clinical development of psychedelics. Arguing against this last concern being definitive, however, is the fact that a minority of participants (13%) reported being harmed by psychedelic use. Cigarette smoking and problematic marijuana use were the most frequently endorsed problems, but a smaller number of respondents believed that their psychedelic use had contributed to suicidal ideation, aggressive/impulsive behavior and/or misuse of benzodiazepines and opiates. It will be important to test the reliability of these findings in subsequent studies and to better determine reasons why individuals feel that psychedelic use contributed to the use of other substances. Not surprisingly, participants who endorsed any of these negative outcomes derived significantly less therapeutic benefit from their psychedelic use, which provides additional face validity to the reality of these harms. However, because we did not enroll a population-based sample, we cannot assume that the low rate of harms reported in the PAWS survey are representative either of the general public or the types of clinical populations that are the target for current psychedelic development programs. Similarly, the PAWS survey did not assess the contexts in which psychedelics were used, so conclusions cannot be drawn regarding the degree to which the occurrence of harms reflect risks of recreational vs. “underground” therapeutic use. As with harms, we are not able to assess the role of dosing context on positive outcomes, which is an important limitation of the current study. Finally, an important limitation of our assessment of harms is that we did not query the occurrence of other potentially serious harmful effects associated with psychedelic use, including the development psychotic reactions and/or Hallucinogen Persisting Perceptual Disorder (HPPD) following psychedelic use or the development of serotonin syndrome symptoms during the dosing period (43, 44). Better characterizing the occurrence of these more specific psychedelic-related negative effects and examining their association with the types of harms identified in the current study will be an important future direction for studies seeking to fully characterize the risk/benefit of these agents. Studies in healthy volunteers and clinical populations consistently find that psychedelics induce acute perceptual/cognitive/emotional states that predict later mental health benefits. In particular, individuals who undergo mystical, emotional breakthrough or psychological insight type experiences during the dosing period are more likely to endorse later benefits ranging from increased personal openness to reductions in depression, anxiety and problematic substance use. Less examined has been the question of whether these types of acute experiences persist and whether their persistence associates with mental health improvements. The PCQ-26 administered in the current study was designed to address this by querying the degree to which mystical, breakthrough and insight experiences common during dosing sessions become habitual states of mind. Results suggest that psychedelics are indeed capable of producing long-term increases in these states and that such increases associate with improvements in mental health. While our design does not allow us to evaluate the causal role of persisting mystical/breakthrough/insight perspectives for mental health, results open the possibility that strategies for optimizing the persistence of these states may enhance the therapeutic benefit of these agents and thereby potentially reduce the need for redosing to maintain clinical effects.

Results from the current study suggest several potentially fruitful lines of future research. Survey-based studies of naturalistic psychedelic use might use prospective designs to more rigorously confirm, or disconfirm, our findings regarding associations between increased frequency of psychedelic use in naturalistic environments and enhanced self-reported wellness. Our results also highlight the importance of looking more exhaustively at the types of harms that naturalistic psychedelic use may engender, as well as their risk factors and impact on long-term well-being and social functioning. In particular, our finding that a minority of participants felt that psychedelic use had increased the problematic use of other substances is an area of obvious importance for the development of these agents as novel therapeutic modalities. Given that naturalistic studies such as ours typically enroll far more heterogenous samples than are allowed within the guard rails of clinical research, they may be especially informative in terms of the risks that will be involved when psychedelics are clinically available to the general population. Finally, we did not observe strong associations between preferred psychedelic agent and self-reported behavioral outcomes, but much remains to be learned from head-to-head comparisons between psychedelics regarding whether one agent is superior to another for any given indication, in either naturalistic or clinical settings.

## Data Availability Statement

The raw data supporting the conclusions of this article will be made available by the authors, without undue reservation.

## Ethics Statement

The studies involving human participants were reviewed and approved by Western Institutional Review Board (WIRB). The patients/participants provided informed consent by clicking an online statement that said, ‘If you agree to participate, please click here to proceed to the survey'.

## Author Contributions

All authors listed have made a substantial, direct, and intellectual contribution to the work and approved it for publication.

## Conflict of Interest

CR is a Consultant for Usona Institute, Otsuka, Novartis, Alfasigma, Emory Healthcare. RJ has received research support, and/or served as a consultant, member of advisory boards, and speaker bureaus of AbbVie (Allergan), Acadia, Adamas, Alfasigma, Alkermes, Axsome, Cingulate Therapeutics, Corium, Eisai, Evidera, Impel, Indivior, Intra-Cellular Therapies, Ironshore Pharmaceuticals, Janssen, Lilly, Lundbeck, Merck, Neos Therapeutics, Neurocrine Biosciences, Osmotica, Otsuka, Pamlab, Pfizer, Sage Therapeutics, Shire, Sunovion, Supernus, Takeda, Teva and Tris Pharmaceuticals. SJ has served as a consultant, member of advisory boards, and/or speaker bureaus for Eli Lily, Otsuka, Pamlab and Sunovion. SC was employed by Research Design Associates. The remaining authors declare that the research was conducted in the absence of any commercial or financial relationships that could be construed as a potential conflict of interest.

## Publisher's Note

All claims expressed in this article are solely those of the authors and do not necessarily represent those of their affiliated organizations, or those of the publisher, the editors and the reviewers. Any product that may be evaluated in this article, or claim that may be made by its manufacturer, is not guaranteed or endorsed by the publisher.
