# Supplementary material for: Effects of Naturalistic Psychedelic Use on Depression, Anxiety, and Well-Being: Associations With Patterns of Use, Reported Harms, and Transformative Mental States
Source: Front Psychiatry. 2022 Mar 15;13:831092. doi: 10.3389/fpsyt.2022.831092 (PMC8965278; doi:10.3389/fpsyt.2022.831092)
Supplement: Supplementary file 1 [file Data_Sheet_1.docx]

**SUPPLEMENTAL INFORMATION**

**Table of Contents**

Psychedelics and Wellness Study (PAWS): An anonymous online survey

exploring the intersect between psychedelics and wellness …………………….………………2

Psychedelic Change Questionnaire (PCQ-26) …………………………………………………23

Negative Consequences Inventory (NCI-8) ……………………………………………………24

Psychedelics and Wellness Study (PAWS): An anonymous online survey exploring the intersect between psychedelics and wellness

**DEMOGRAPHICS:**

Have you ever taken a psychedelic?

 YES

 NO

If NO, message: Thank you for your interest in participating in this anonymous survey. However, you are not eligible to participate since you have never taken a psychedelic.

If YES, next question.

Age (must be 18 years or older): Drop down menu, ages 18-100

Gender:

 MALE

 FEMALE

 OTHER

Education:

 Less than high school degree

 High school degree or equivalent (e.g., GED)

 Some college but no degree

 Associate degree

 Bachelor’s degree

 Graduate degree

 Professional degree

What is your psychedelic of choice (the one you believe to be most beneficial)?

PLEASE ONLY SELECT ONE

 Psilocybin (magic mushrooms)

 LSD

 Ayahuasca

 Mescaline/ Peyote/San Pedro/Other Mescaline containing cacti

 5-MeO-DMT

 Ketamine

 Iboga/Ibogaine

 2C-B

 2C-E

 DMT

 Salvia

 Other designer/synthetic psychedelics

What is the total number of times you have taken any psychedelic in your lifetime? Drop down menu, options 1-500

Have you ever micro-dosed a psychedelic substance?

 YES

 NO

Are you a healthcare provider (such as an MD, DO, NP, or PA) who treats psychiatric disorders with medications?

 YES

 NO

**Please answer the following questions based on how you felt PRIOR TO EVER TAKING a psychedelic in your lifetime.**

HERO Wellness Scale (Pre)


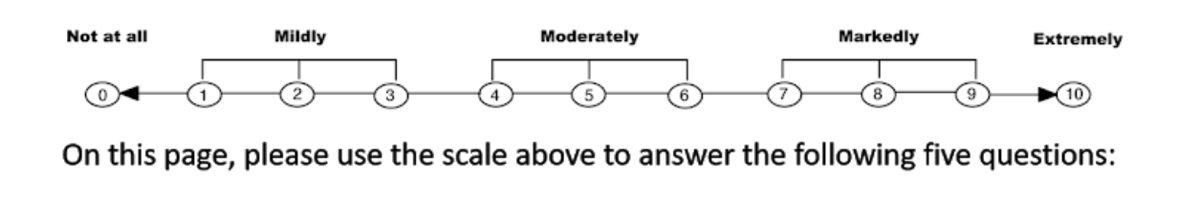


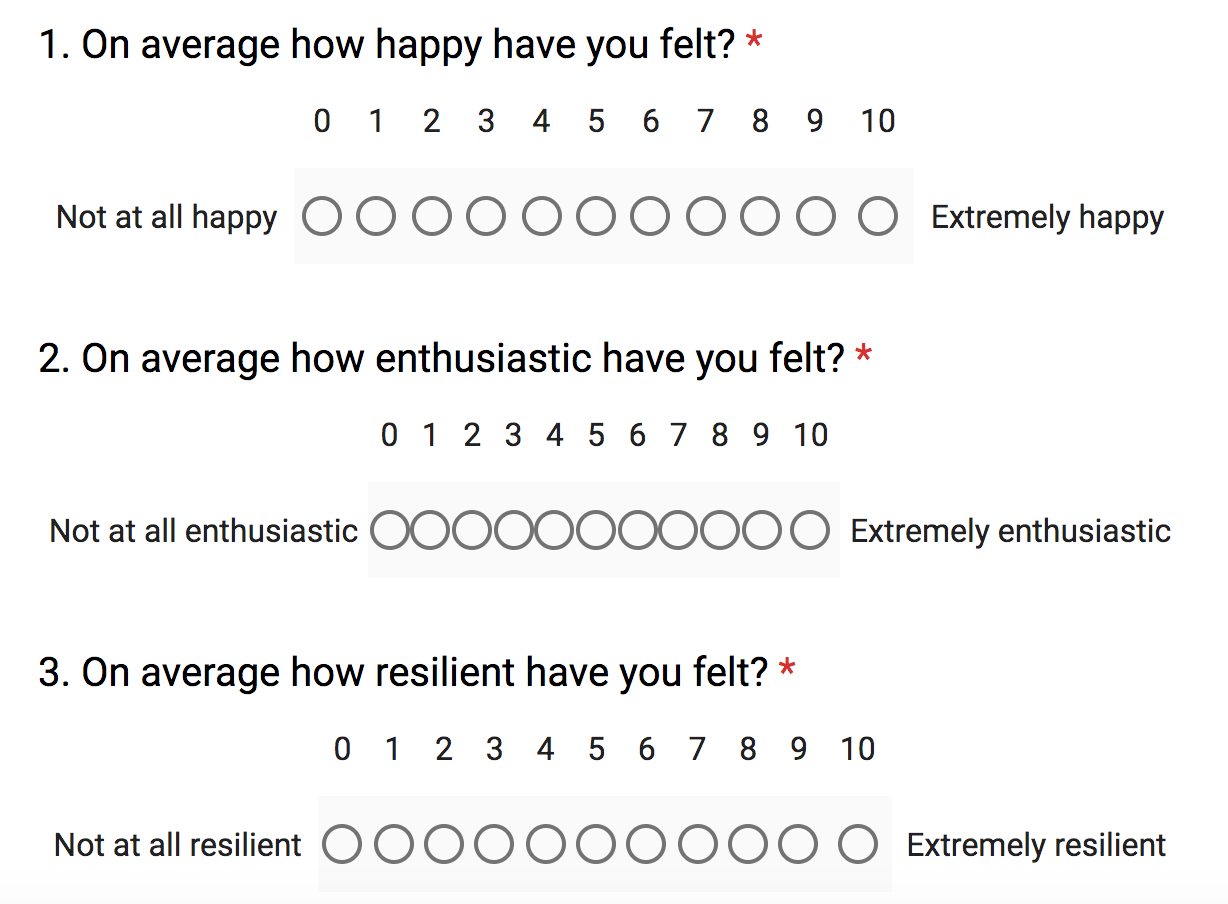


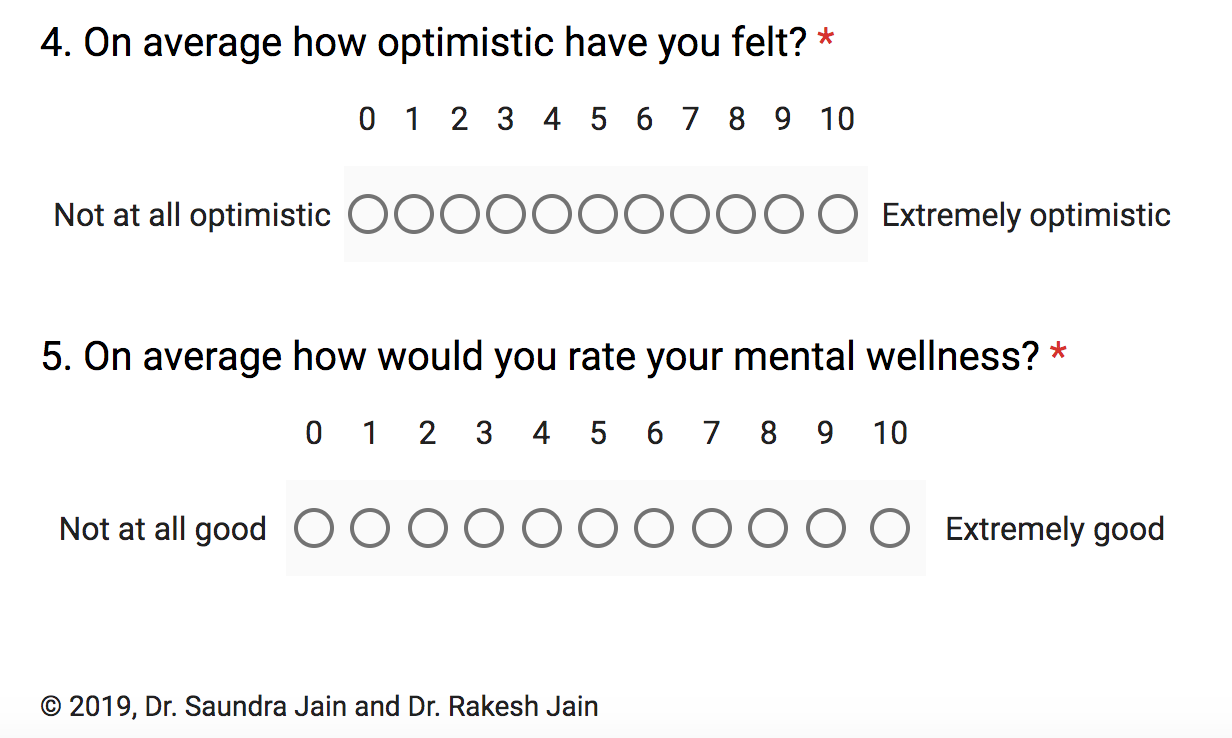


**Please answer the following questions based on how you felt PRIOR TO EVER TAKING a psychedelic in your lifetime.**

Patient Health Questionnaire-9 (Pre)


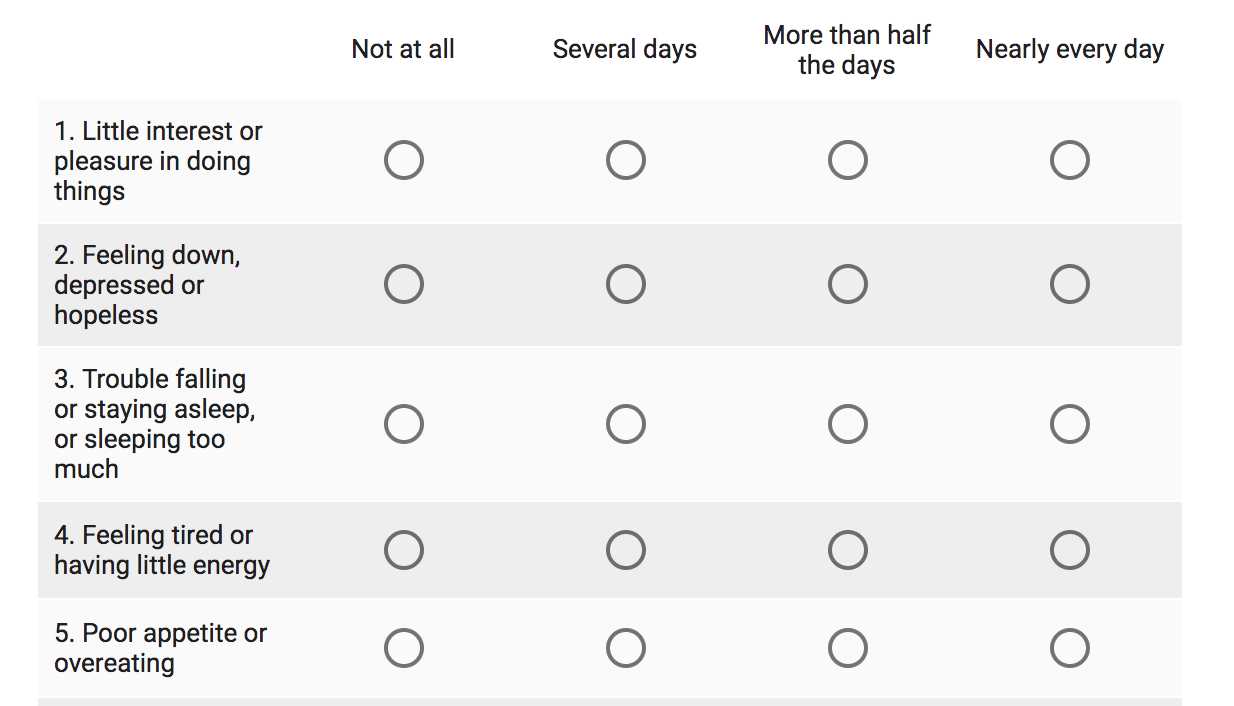


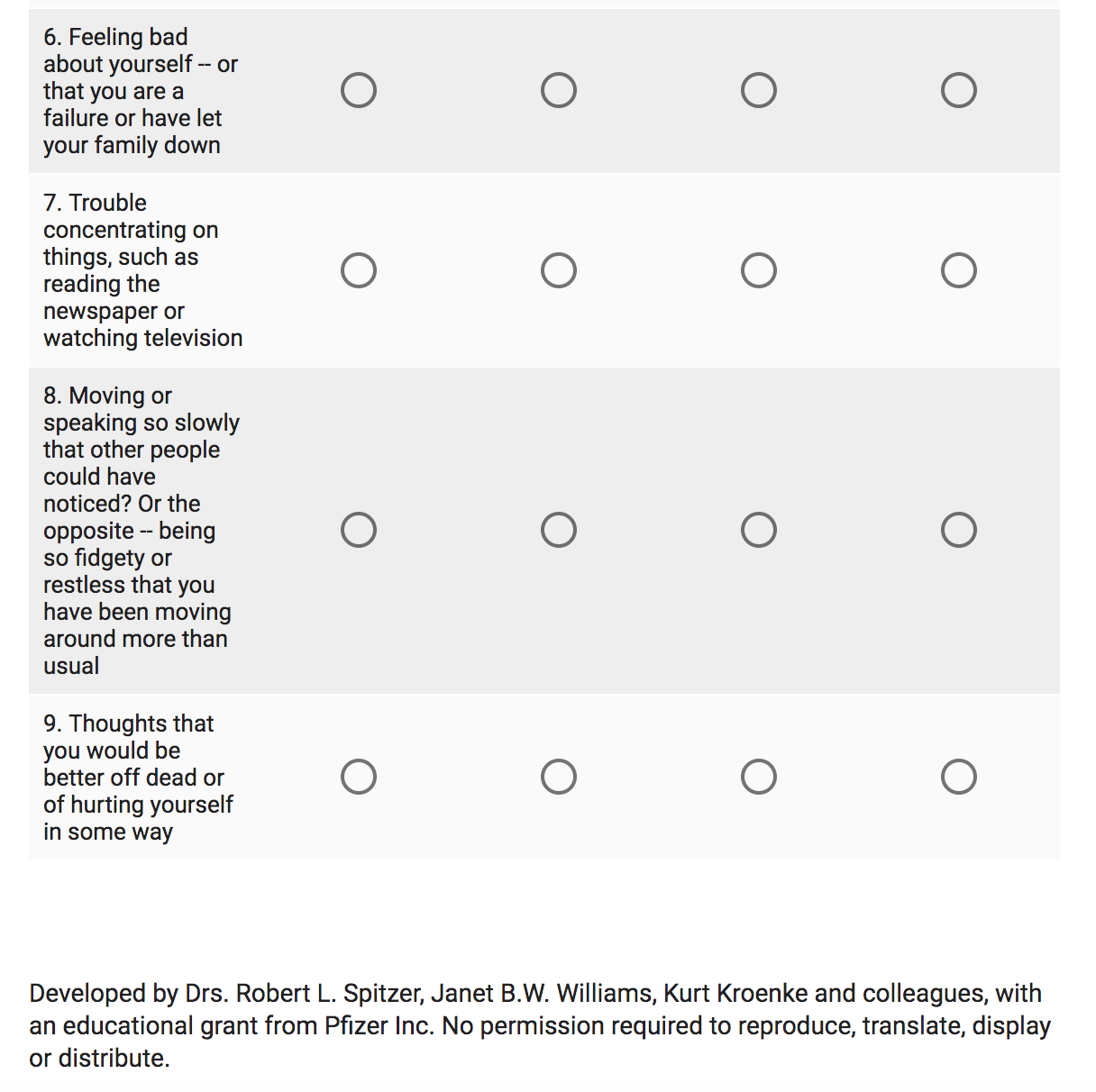


**Please answer the following questions based on how you felt PRIOR TO EVER TAKING a psychedelic in your lifetime.**

Generalized Anxiety Disorder-7 (Pre)


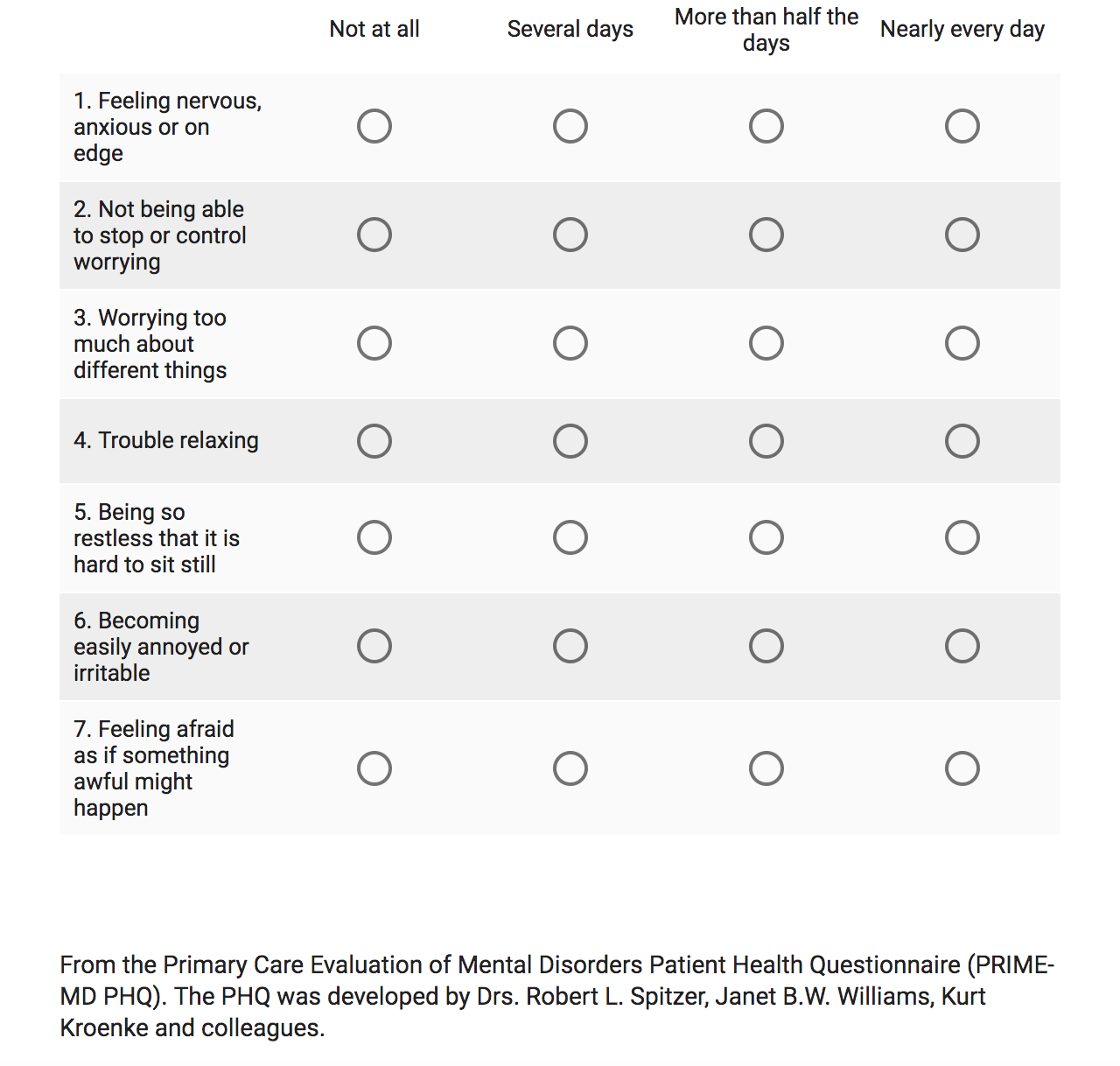


**Please answer the following questions based on your overall sense of wellness AS A RESULT or your psychedelic experience(s).**

HERO Wellness Scale (Post)


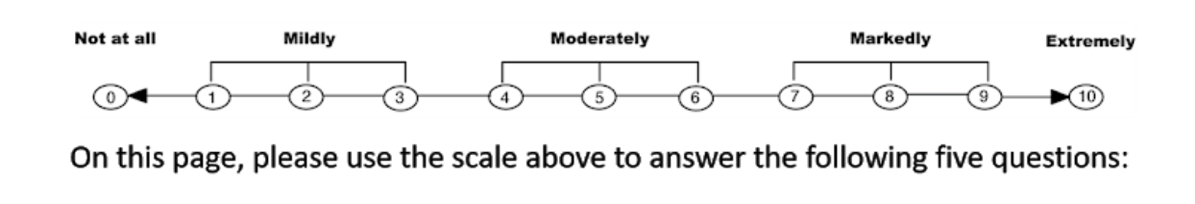


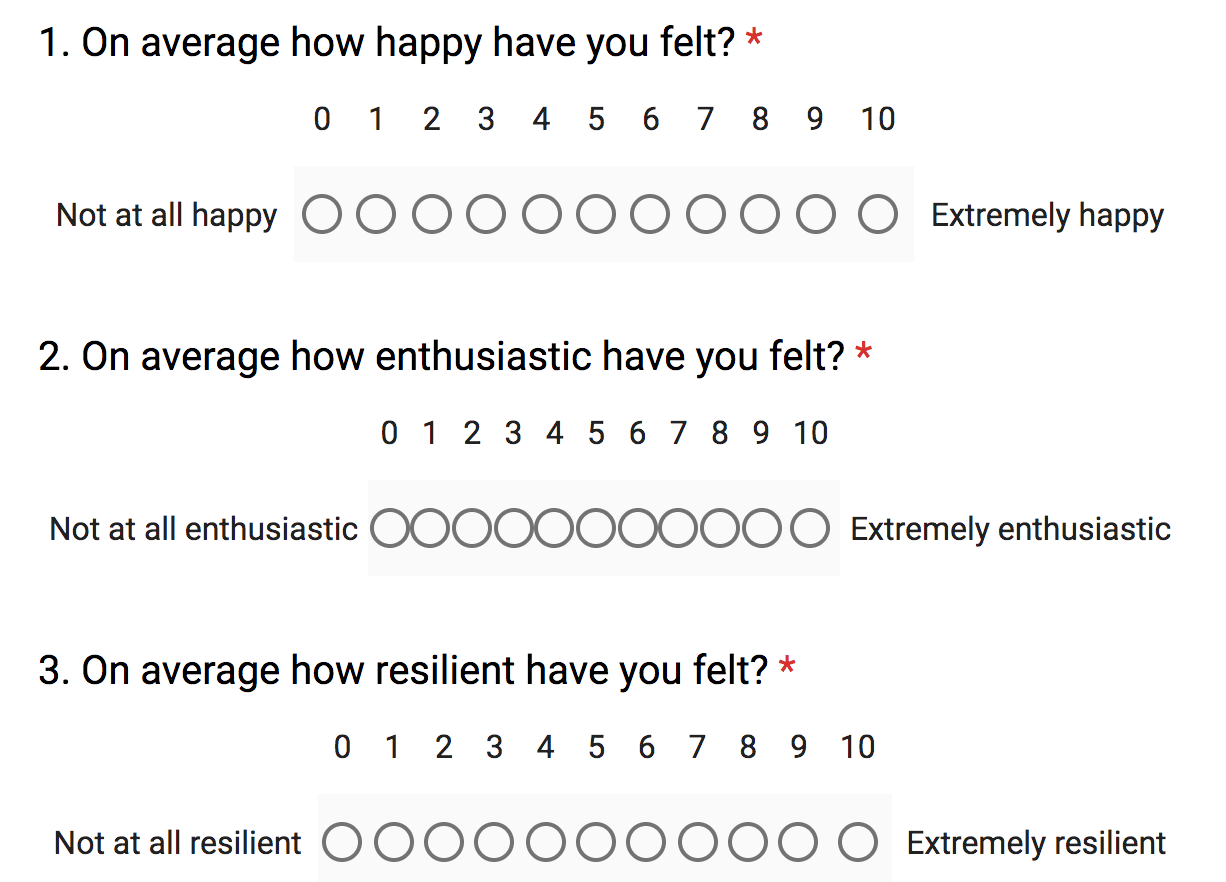


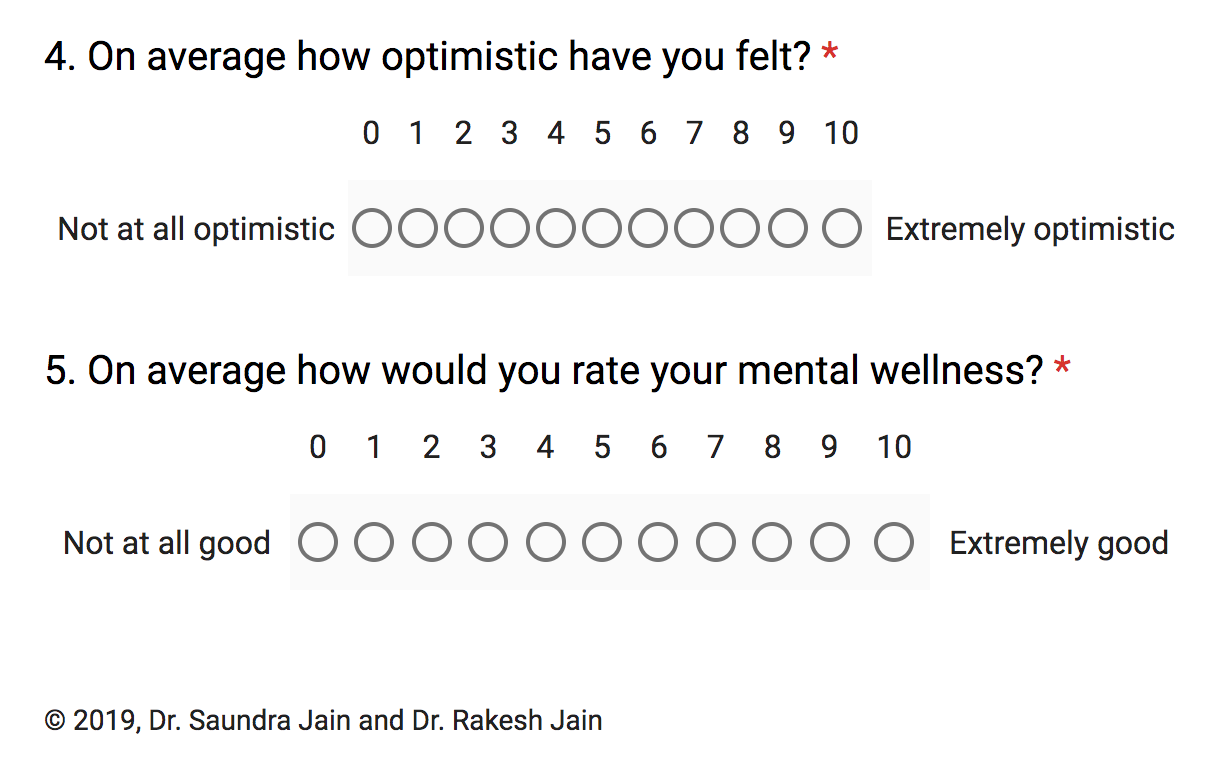


**Please answer the following questions based on your overall mood AS A RESULT or your psychedelic experience(s).**

Patient Health Questionnaire-9 (Post)


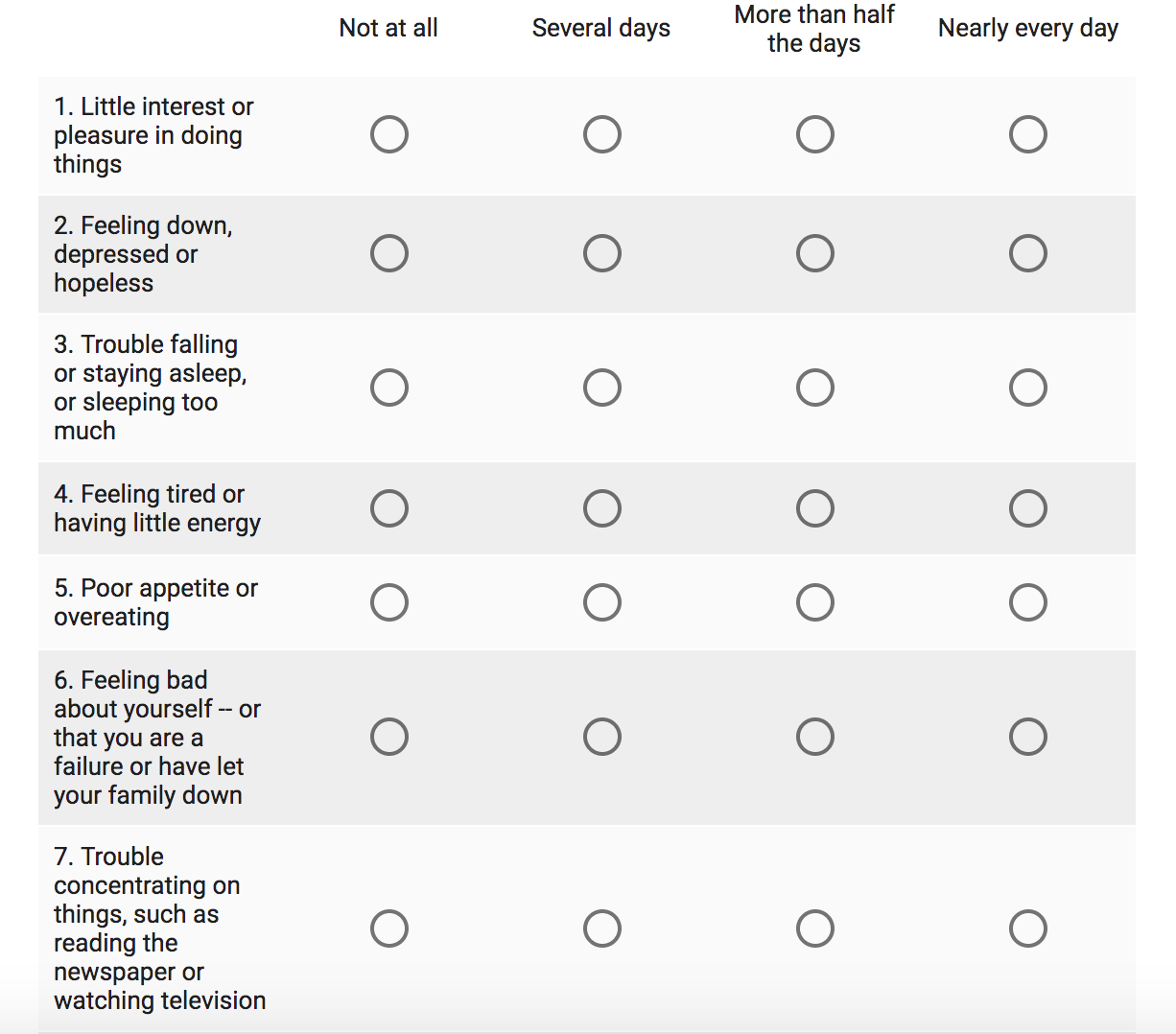


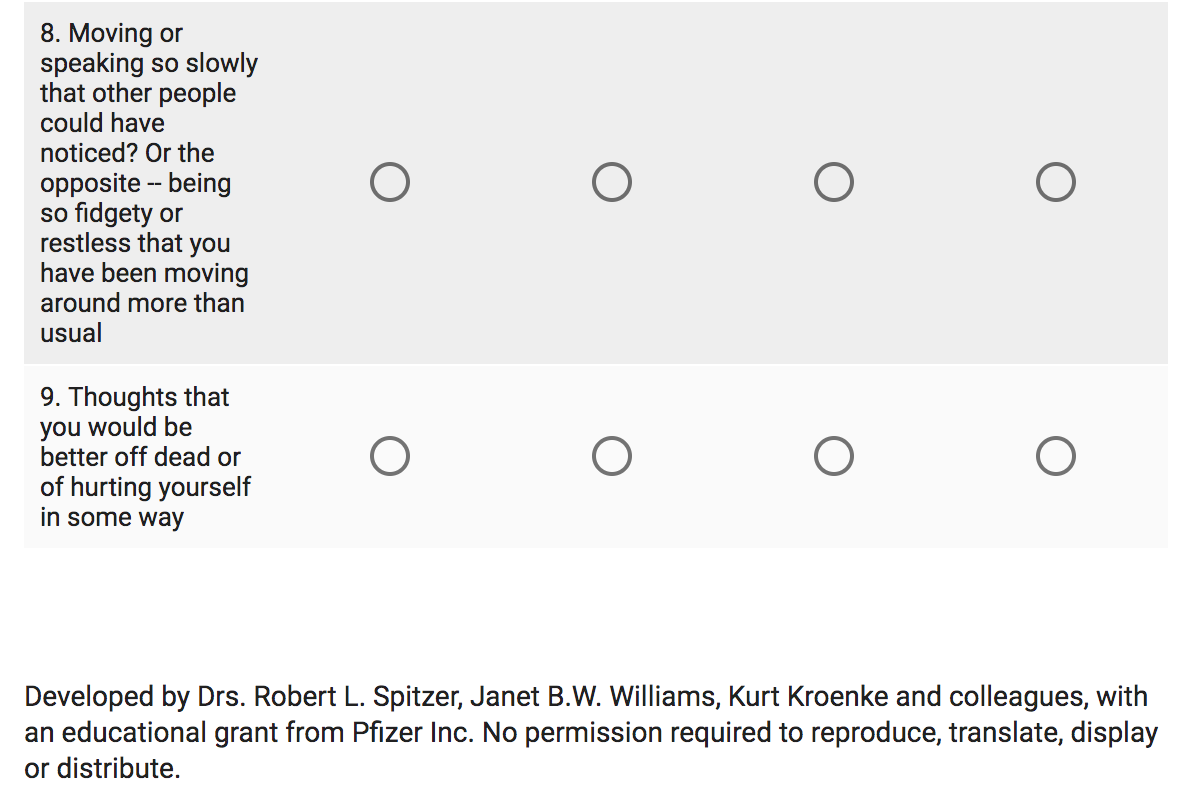


**Please answer the following questions based on your overall mood AS A RESULT or your psychedelic experience(s).**

Generalized Anxiety Disorder (Post)


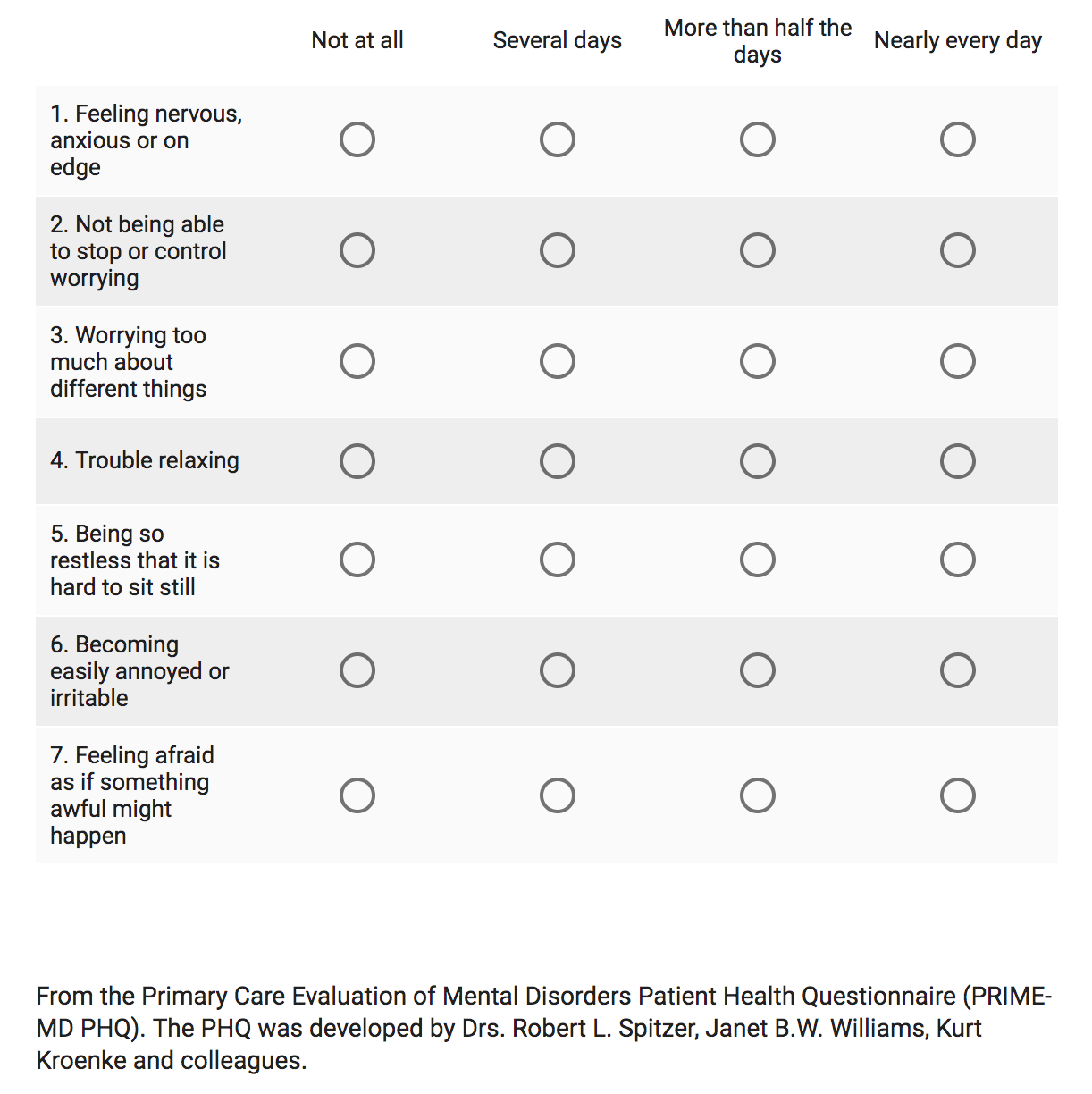


**Please answer the following questions by rating the CHANGE in your experiences, feelings, and behaviors AS A RESULT of your psychedelic experience(s).**

1. As a result of your psychedelic experience(s), how would you rate the change in your SENSE OF AWE?


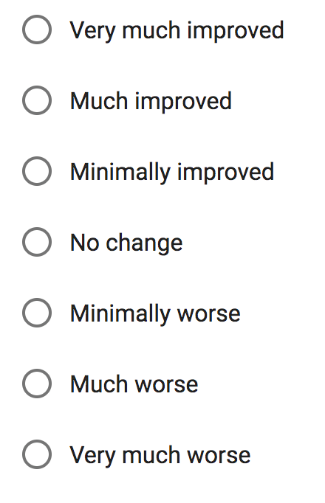


2. As a result of your psychedelic experience(s), how would you rate the change in your FEELINGS OF EMPATHY?


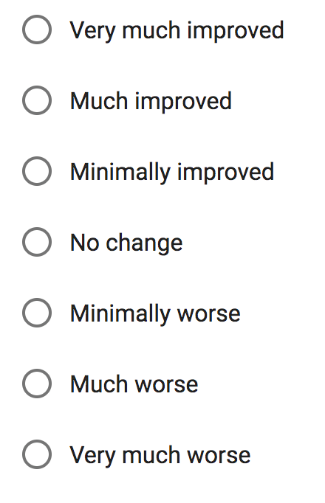


3. As a result of your psychedelic experience(s), how would you rate the change in your FEELINGS OF SOCIAL CONNECTEDNESS?


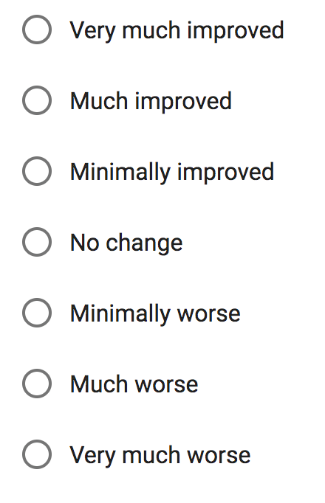


4. As a result of your psychedelic experience(s), how would you rate the change in your SENSE OF MINDFULNESS?


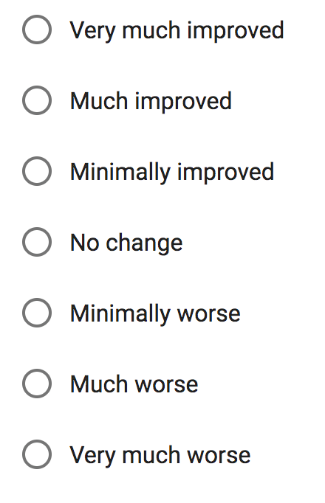


5. As a result of your psychedelic experience(s), how would you rate the change in your RELATIONSHIP WITH YOUR LIFE PARTNER?


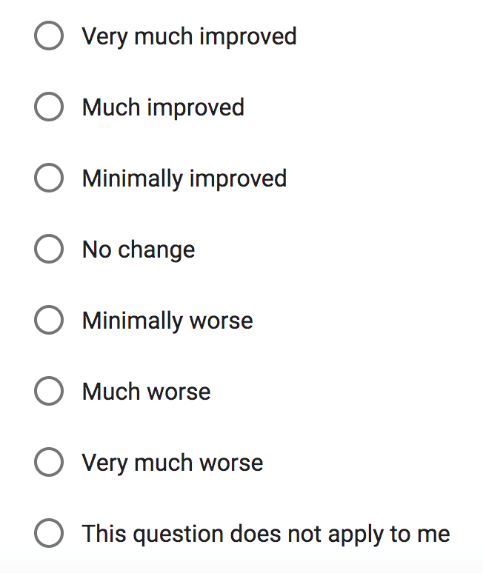


6. As a result of your psychedelic experience(s), how would you rate the change in your FEELINGS OF SEXUAL INTIMACY?


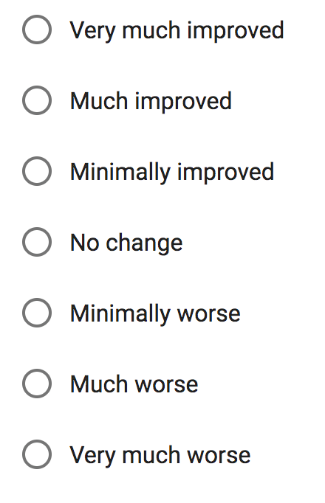


7. As a result of your psychedelic experience(s), how would you rate the change in your QUALITY OF SLEEP?


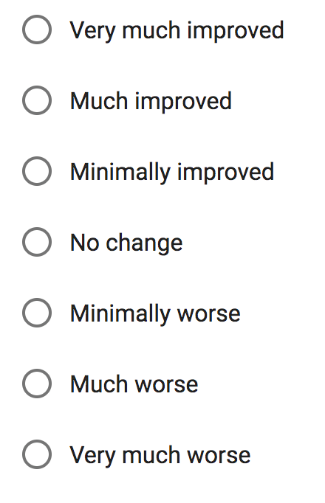


8. As a result of your psychedelic experience(s), how would you rate the change in your FEELINGS OF INNER PEACE?


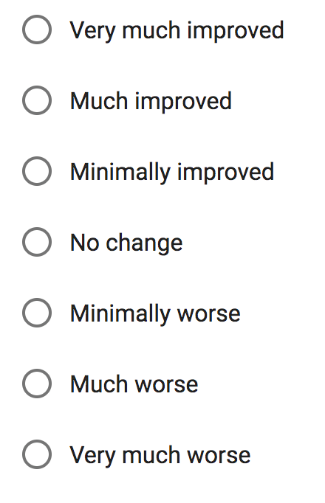


9. As a result of your psychedelic experience(s), how would you rate the change in your SENSE OF CALM?


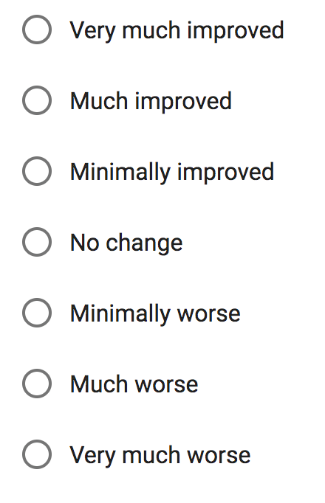


10. As a result of your psychedelic experience(s), how would you rate the change in your CONNECTION TO THE UNIVERSE?


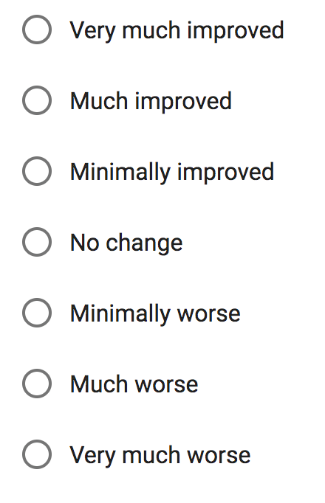


11. As a result of your psychedelic experience(s), how would you rate the change in your CONNECTION TO NATURE?


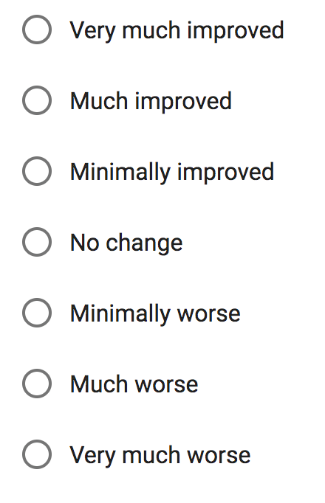


12. As a result of your psychedelic experience(s), how would you rate the change in your FEELINGS OF LOVE?


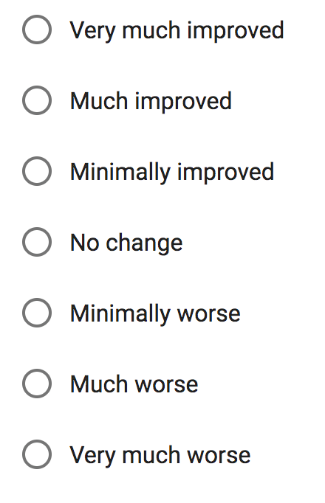


13. As a result of your psychedelic experience(s), how would you rate the change in your FEELINGS OF JOY?


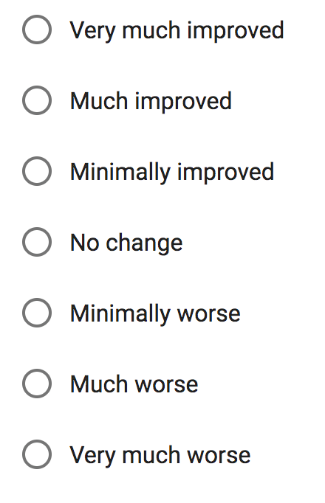


14. As a result of your psychedelic experience(s), how would you rate the change in your FEELINGS OF OPENESS?


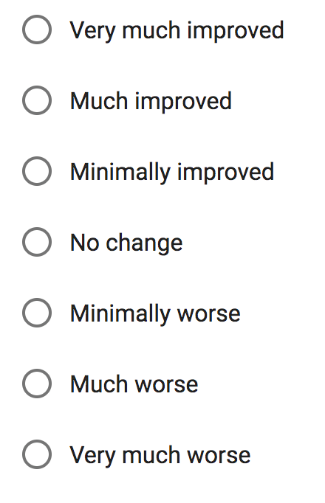


15. As a result of your psychedelic experience(s), how would you rate the change in your FEELINGS OF CONTENTMENT?


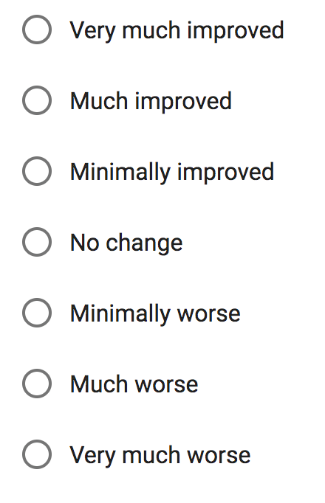


16. As a result of your psychedelic experience(s), how would you rate the change in your FEELINGS OF GRATITUDE?


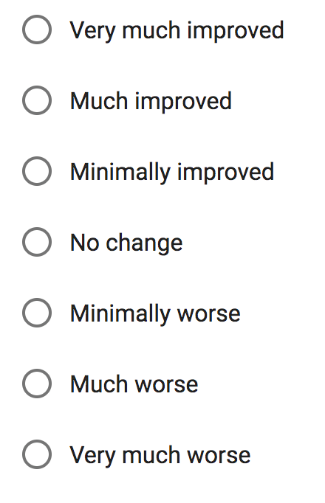


17. As a result of your psychedelic experience(s), how would you rate the change in your SENSE OF PURPOSE?


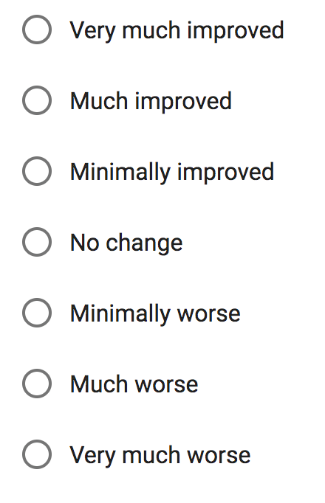


18. As a result of your psychedelic experience(s), how would you rate the change in your FEELINGS OF COMPASSION?


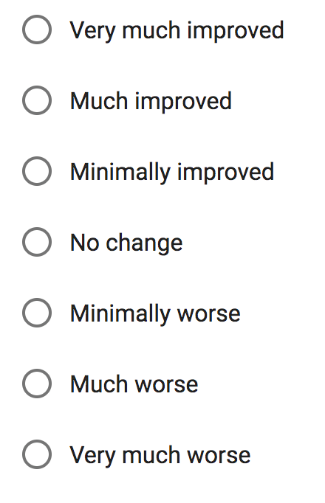


19. As a result of your psychedelic experience(s), how would you rate the change in your DESIRE FOR WORLD PEACE?


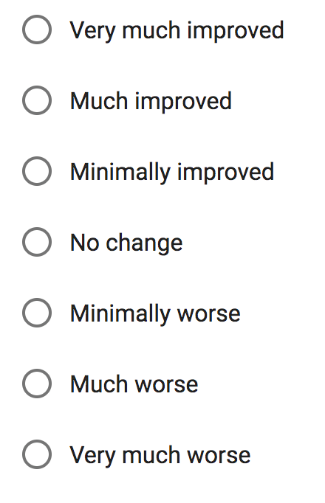


20. As a result of your psychedelic experience(s), how would you rate the change in your ENJOYMENT OF LIFE?


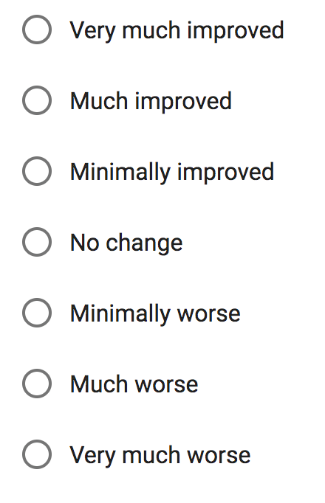


21. As a result of your psychedelic experience(s), how would you rate the change in your ALTRUISTIC DESIRE (the belief in or practice of selfless concern for the well-being of others)?


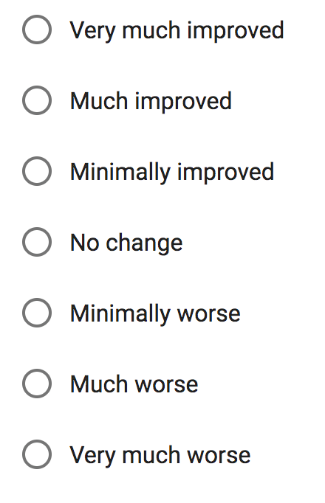


22. As a result of your psychedelic experience(s), how would you rate the change in your PHILANTHROPIC DESIRE (defined as donation of money or service to serve the needs of others)?


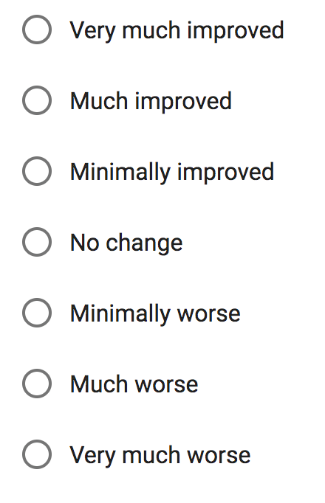


23. As a result of your psychedelic experience(s), how would you rate the change in your RUMINATIVE THINKING (compulsive focus of attention on thoughts that cause feelings of sadness, anxiety, distress, etc.)?


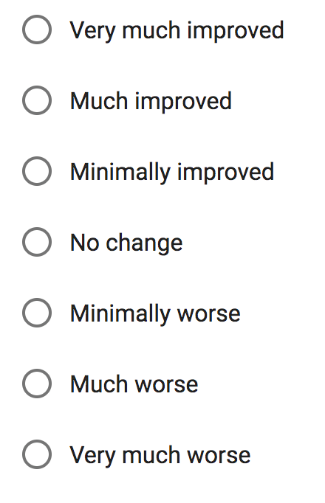


24. As a result of your psychedelic experience(s), how would you rate the change in your FEAR OF DEATH?


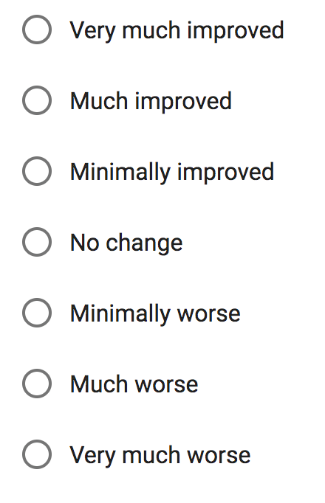


25. As a result of your psychedelic experience(s), how would you rate the change in your EATING HABITS?


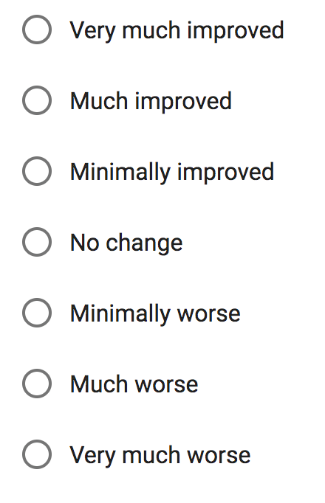


26. As a result of your psychedelic experience(s), how would you rate the change in your FEELINGS OF IRRITABILITY?


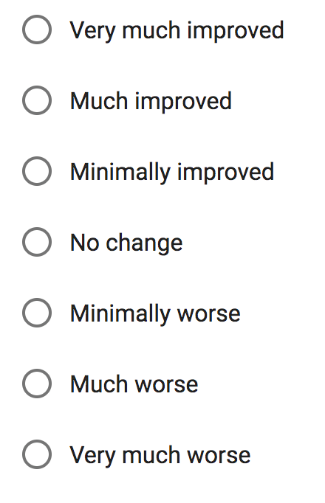


27. As a result of your psychedelic experience(s), how would you rate the change in your DESIRE TO DIE BY SUICIDE?


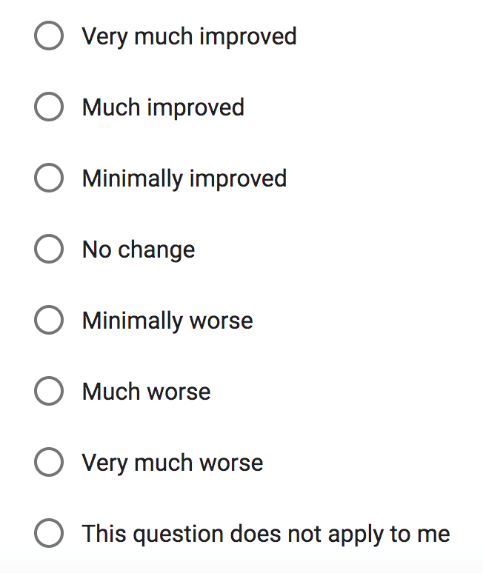


28. As a result of your psychedelic experience(s), how would you rate the change in your CRIMINAL IMPULSES/BEHAVIORS?


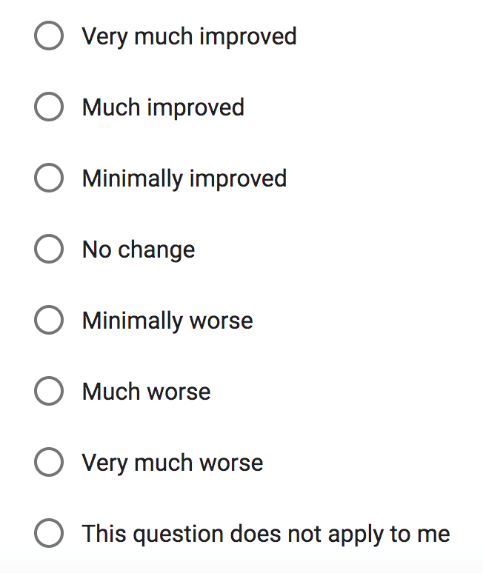


29. As a result of your psychedelic experience(s), how would you rate the change in your AGGRESSIVE IMPULSES/BEHAVIORS?


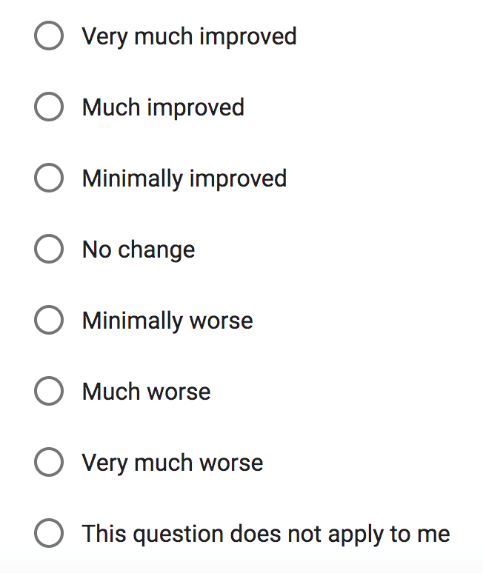


30. As a result of your psychedelic experience(s), how would you rate the change in your ALCOHOL MISUSE?


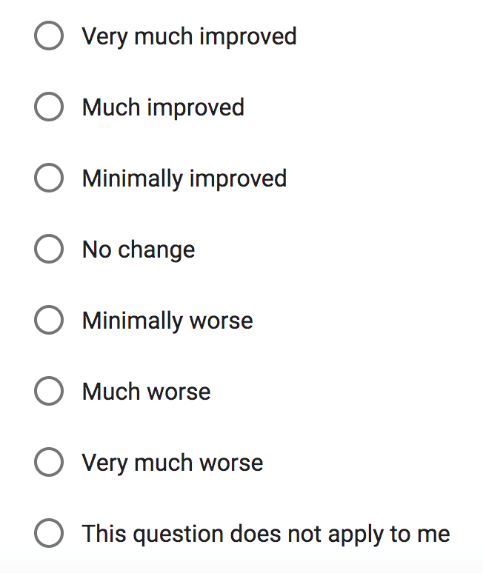


31. As a result of your psychedelic experience(s), how would you rate the change in your CIGARETTE SMOKING?


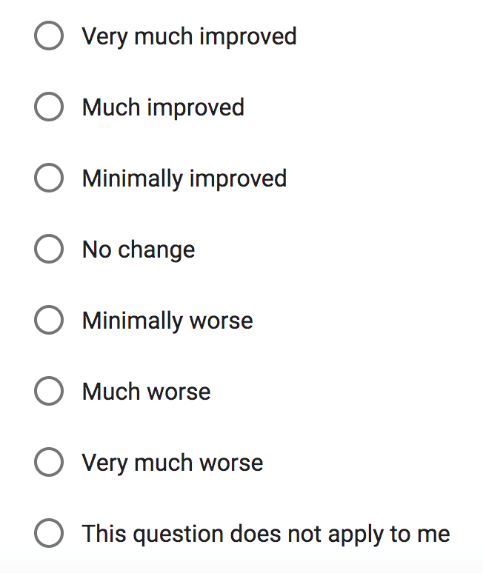


32. As a result of your psychedelic experience(s), how would you rate the change in your CANNABIS/MARIJUANA MISUSE?


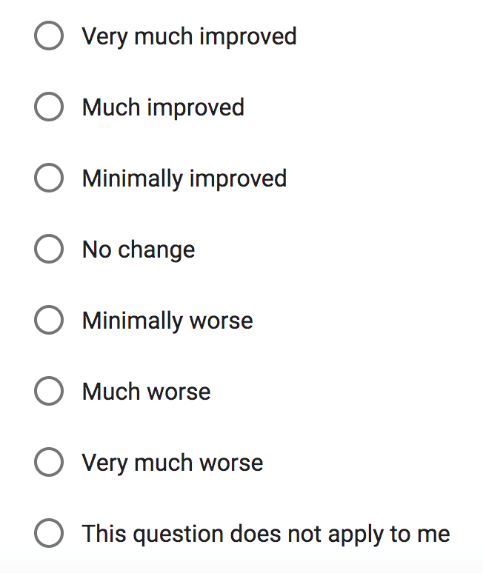


33. As a result of your psychedelic experience(s), how would you rate the change in your BENZODIAZEPINE MISUSE?


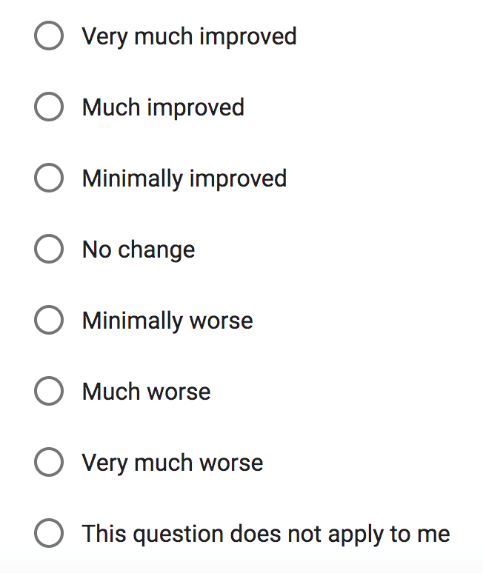


34. As a result of your psychedelic experience(s), how would you rate the change in your OPIATE/OPIOID MISUSE?


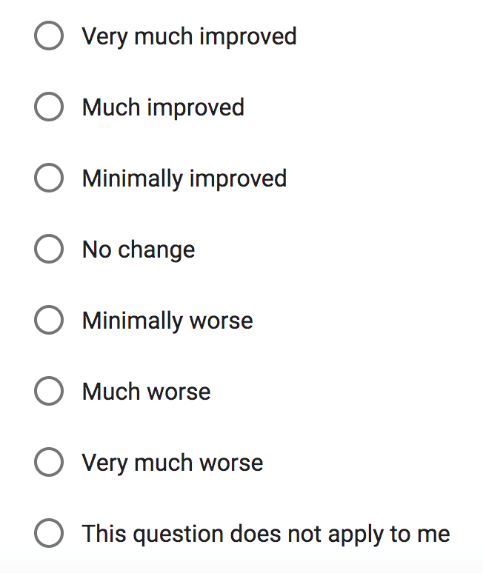


**The next few questions pertain to your opinion regarding addiction, legalization, and medical use of psychedelics.**

1. Do you believe that psychedelics are addictive substances?

YES

NO

2. Do you believe psychedelics should be used to treat certain psychiatric disorders (such as major depression and post-traumatic stress disorder)?

YES

NO

3. Do you believe psychedelics should be legalized for medical use?

YES

NO

**Please CICK THE SUBMIT BUTTON to record your answers. Thank you, again, for taking part in this study – your input is greatly appreciated!**

**Mental Health Resources**

Participation in this study does not imply any type of professional relationship with the researchers. If you are in crisis, please contact your healthcare provider or refer to the list of resources below:

- National Suicide Prevention Lifeline – 800.273.TALK (8255). This is a 24-hour, toll-free, confidential suicide prevention hotline available to anyone in suicidal crisis or emotional distress.
- Crisis Text Line – 741741 (Text “HOME”). This is a 24-hour, free, support of persons in any type of crisis, including suicidal ideation, via text medium. <https://www.crisistextline.org/texting-in>
- SAMHSA (Substance Abuse and Mental Health Services Administration) National Helpline – 800.662.HELP (4357). This is a free, confidential, 24/7, 365-day-a-year treatment referral and information service (in English and Spanish) for individuals and families facing mental health and/or substance use disorders.
- 911 - This number is intended for use in emergency circumstances only.

**Psychedelic Change Questionnaire (PCQ-26)**

**Please answer the following questions by rating the CHANGE in your experiences, feelings, and behaviors as a result of your MOST RECENT psychedelic experience.**

|  | **1** | **2** | **3** | **4** | **5** | **6** | **7** |
| --- | --- | --- | --- | --- | --- | --- | --- |
|  | **Very much improved** | **Much improved** | **Minimally improved** | **No**  **change** | **Minimally worse** | **Much Worse** | **Very much worse** |
| **As a result of your psychedelic experience, how would you rate the change in your …** | | | | | | | |
| 1. … SENSE OF AWE? |  |  |  |  |  |  |  |
| 1. … FEELINGS OF EMPATHY? |  |  |  |  |  |  |  |
| 1. … FEELINGS OF SOCIAL CONNECTEDNESS? |  |  |  |  |  |  |  |
| 1. … SENSE OF MINDFULNESS? |  |  |  |  |  |  |  |
| 1. … RELATIONSHIP WITH YOUR LIFE PARTNER? |  |  |  |  |  |  |  |
| 1. … FEELINGS OF SEXUAL INTIMACY? |  |  |  |  |  |  |  |
| 1. … QUALITY OF SLEEP? |  |  |  |  |  |  |  |
| 1. … FEELINGS OF INNER PEACE? |  |  |  |  |  |  |  |
| 1. … SENSE OF CALM? |  |  |  |  |  |  |  |
| 1. … CONNECTION TO THE UNIVERSE? |  |  |  |  |  |  |  |
| 1. … CONNECTION TO NATURE? |  |  |  |  |  |  |  |
| 1. … FEELINGS OF LOVE? |  |  |  |  |  |  |  |
| 1. … FEELINGS OF JOY? |  |  |  |  |  |  |  |
| 1. … FEELINGS OF OPENESS? |  |  |  |  |  |  |  |
| 1. … FEELINGS OF CONTENTMENT? |  |  |  |  |  |  |  |
| 1. … FEELINGS OF GRATITUDE? |  |  |  |  |  |  |  |
| 1. … SENSE OF PURPOSE? |  |  |  |  |  |  |  |
| 1. … FEELINGS OF COMPASSION? |  |  |  |  |  |  |  |
| 1. … DESIRE FOR WORLD PEACE? |  |  |  |  |  |  |  |
| 1. … ENJOYMENT OF LIFE? |  |  |  |  |  |  |  |
| 1. … ALTRUISTIC DESIRE? *(the belief in or practice of selfless concern for the well-being of others)* |  |  |  |  |  |  |  |
| 1. … PHILANTHROPIC DESIRE? *(defined as donation of money or service to serve the needs of others)* |  |  |  |  |  |  |  |
| 1. … RUMINATIVE THINKING? *(compulsive focus of attention on thoughts that cause feelings of sadness, anxiety, distress, etc.)* |  |  |  |  |  |  |  |
| 1. … FEAR OF DEATH? |  |  |  |  |  |  |  |
| 1. … EATING HABITS? |  |  |  |  |  |  |  |
| 1. … FEELINGS OF IRRITABILITY? |  |  |  |  |  |  |  |

**Negative Consequences Inventory (NCI-8)**

**Please answer the following questions by rating the CHANGE in your experiences, feelings, and behaviors as a result of your MOST RECENT psychedelic experience.**

|  | **1** | **2** | **3** | **4** | **5** | **6** | **7** | **Does not apply** |
| --- | --- | --- | --- | --- | --- | --- | --- | --- |
|  | **Very much improved** | **Much improved** | **Minimally improved** | **No**  **change** | **Minimally worse** | **Much Worse** | **Very much worse** |  |
| **As a result of your psychedelic experience, how would you rate the change in your …** | | | | | | | | |
| 1. … DESIRE TO DIE BY SUICIDE? |  |  |  |  |  |  |  |  |
| 1. … CRIMINAL IMPULSES/BEHAVIORS? |  |  |  |  |  |  |  |  |
| 1. … AGGRESSIVE IMPULSES/BEHAVIORS? |  |  |  |  |  |  |  |  |
| 1. … ALCOHOL MISUSE? |  |  |  |  |  |  |  |  |
| 1. … CIGARETTE SMOKING? |  |  |  |  |  |  |  |  |
| 1. … CANNABIS/MARIJUANA MISUSE? |  |  |  |  |  |  |  |  |
| 1. … BENZODIAZEPINE MISUSE? |  |  |  |  |  |  |  |  |
| 1. … OPIATE/OPIOID MISUSE? |  |  |  |  |  |  |  |  |
